# Key biomarkers within the colorectal cancer related inflammatory microenvironment

**DOI:** 10.1038/s41598-021-86941-5

**Published:** 2021-04-12

**Authors:** Valentin Calu, Adriana Ionescu, Loredana Stanca, Ovidiu Ionut Geicu, Florin Iordache, Aurelia Magdalena Pisoschi, Andreea Iren Serban, Liviu Bilteanu

**Affiliations:** 1 Department of General Surgery, University of Medicine and Pharmacy “Carol Davila” Bucharest, 8 Blvd., Eroii Sanitari, 050474 Bucharest, Romania; 2 Department of Surgery, “Elias” Emergency University Hospital, 17 Marasti Blvd., 01146 Bucharest, Romania; 3 Department of Biochemistry and Molecular Biology, Faculty of Biology, University of Bucharest, 91-95 Blvd. Splaiul Independentei, 050095 Bucharest, Romania; 4 Department of Preclinic Sciences, Faculty of Veterinary Medicine, University of Agronomic Sciences and Veterinary Medicine of Bucharest, 105 Blvd. Splaiul Independentei, 050097 Bucharest, Romania; 5 Taxon Solutions SRL, 7 Semilunei Str, 020797 Bucharest, Romania; 6 National Institute for Research and Development in Microtechnologies, 126A Erou Iancu Nicolae Street, 077190 Bucharest, Romania

**Keywords:** Biomarkers, Medical research, Molecular medicine, Oncology

## Abstract

Therapeutic approaches focused on the inflammatory microenvironment are currently gaining more support, as biomolecules involved in the inflammatory colorectal cancer (CRC) tumor microenvironment are being explored. We analyzed tumor and paired normal tissue samples from CRC patients (n = 22) whom underwent tumor resection surgery. We assessed 39 inflammation-involved biomolecules (multiplex magnetic bead-based immunoassay), CEA and CA19-9 (ELISA assay) and the tissue expression levels of occludin and also pErk, STAT1 and STAT3 transcriptional factors (western blot). Tumor staging has been established by histopathological evaluation of HE stained tumor tissue sections. We report 32 biomarkers displaying statistically significant differences in tumor vs. control. Additionally, positive statistical biomarker correlations were found between MMP2–IL8 and BAFF–IL8 (Pearson correlation coefficients > 0.751), while APRIL–MMP2, APRIL–BAFF and APRIL–IL8 were negatively correlated (correlation coefficients < − 0.650). While APRIL, BAFF, IL8 and MMP2 did not modulate with tumor stage, they were inversely related to the immune infiltrate level and CD163 tissue expression. We conclude that the significantly decreased APRIL and increased BAFF, IL8 and MMP2 expression were tumor-specific and deserve consideration in the development of new treatments. Also, the positive correlation between Chitinase 3-like 1 and IL8 (0.57) or MMP2 (0.50) suggest a role in tumor growth and metastasis pathways.

## Introduction

Colorectal cancer (CRC) is the third most common fatal malignancy worldwide^1^, almost 50% of these patients eventually succumb to the disease. In recent years, there have been major advances in understanding the molecular basis of tumors and their progression from adenoma to carcinoma. These pathways can hold potential for developing novel strategies for the treatment of CRC^2^. The malignant phenotype activates inflammatory cells leading to cellular immunity alteration and forcing these cells to produce soluble factors (cytokines, chemokines, growth factors, proteases, etc.) that regulate growth, differentiation and survival of tumor cells. Tumor expansion causes thus significant peritumoral inflammation which determines the continuous activation of the pathways involved in tumor initiation, promotion and progression^3^. These stages of neoplastic evolution are enhanced in the inflammatory context which promotes sustained proliferation, resistance to apoptosis, reprogramming and reorganization of the stromal environment, and genomic instability^4^. In addition, tumor cells increase the inflammatory constellation by producing a wide range of cytokines, cytotoxic mediators including reactive oxygen species, serine and cysteine proteases, MMPs and membrane-perforating agents^4,5^. In order to modulate the immune response towards cancer cells and induce, if possible, tumor cell death, cytokines, which are a part of the immune response, are targeted in some types of immunotherapies. However, focusing on a single cytokine was shown to have only moderate efficiency in low doses, while high dose monotherapies led to significant side effects. The development of multi-targeted immunotherapies requires a thorough understanding of correlated cytokines actions within the anti-tumor response^6^. This led to investigations of extended panels of inflammatory markers in cancer patients. Molecules such as IL-20, IL-1β and IL-22 are currently being considered as potential therapeutic targets in CRC^7^. Several studies employing extensive multiplex assays for inflammatory profiling led to a wide diversity of encouraging results, for instance IL-6^8–11^ and IL-8^10–12^, expressions are higher in serum patients with CRC, and are currently being considered as candidates in immunotherapies. Other biomarkers with diagnostic and prognostic value in CRC reported by several studies are IL-8 programmed death ligand 1 (PD-L1)^10^, CEA^11,13^, CA19-9^12^, MMP-9^13^ etc. Despite such a multitude of studies, to date there is no unique panel of biomarkers capable to distinguish between different types of cancers and more studies are needed to find a reliable panel of biomarkers in terms of sensitivity and specificity for predictive and diagnostic purposes. Moreover, a combination of these markers could improve the understanding of the complex chain of molecular phenomena leading to this phenotype.

In this work, we describe through statistical correlations some possible molecular pathways involving tumor-related inflammation markers and their association to CRC progression. Molecules that are simultaneously involved in CRC progression through common pathways might be either eligible targets for new immunotherapy drugs (aiming to improve life quality and lifespan of CRC patients) or markers in a screening panel that will monitor CRC patients evolution and improve of post-surgical care.

## Results

### Array data

The level of 39 inflammatory molecules in tumor and paired normal tissue collected from CRC patients during tumor resection surgery have been quantified using the Bio-Plex Pro assay. In addition, the concentrations of CEA and CA19-9 were detected by ELISA, while the protein expression levels of phosphorylated extracellular‑regulated kinase ½ (pErk1/2), occludin (OCLN), signal transducer and activator of transcription 1 (STAT1) and 3 (STAT3), and carbonylated proteins (CP) were assessed through immunobloting. The concentrations of the following biomarkers (in alphabetical order) have been under the lower limit of quantification (LLOQ) or under the limit of detection (LOD): IFN-α2, IL-2, IL-10, IL-11, IL-12 (p40), IL-12 (p70), IL-19, IL-27 (p28), IL-28A/IFN-λ2, IL-29/IFN-λ1 and osteocalcin.

**Figure 1:**
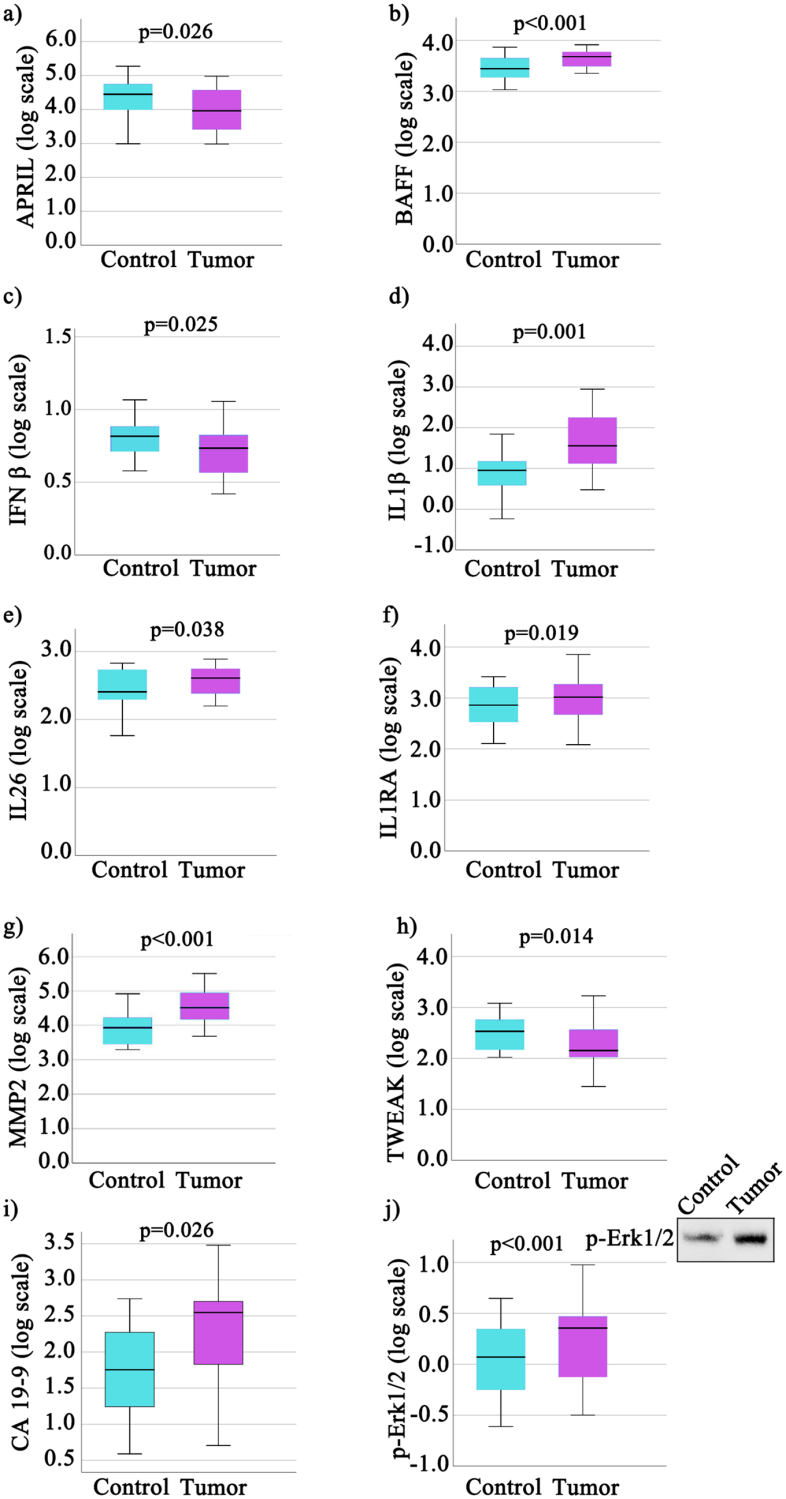
Box-plots of log-transformed biomarker values in tumor and paired normal tissue samples. Selected log-transformed biomarker values had a normal distribution. (**a**) APRIL; (**b**) BAFF; (**c**) IFN β; (**d**) IL1β; (**e**) IL26; (f) IL1RA; (**g**) MMP2; (**h**) TWEAK; (**i**) CA19-9; (**j**) p-Erk1/2. In box-plot (**j**) the source data was the average relative density of immunoblot bands of all tumor and paired normal tissue samples; the cropped insert shows a representative immunoblot example. The original immunoblots are available in Supplementary Figure S1; *p* values resulting from comparison of control and tumor means of log-transformed levels through t Student test are written above each boxplot pair.

All the remaining raw data sets detected beyond LOD (see Supplementary Table S1), have failed the initial Shapiro-Wilks test. After log-transformation, the following biomarkers displayed a normal distribution and statistically significant differences in tumor vs. control (in alphabetical order): APRIL, BAFF, CA19-9, pERK, IL-1β, IL-1RA, IFN-β, IL-26, MMP-2, and TWEAK (Fig. 1). Showing similar distribution as in the previous series, TSLP exhibited downregulation when comparing its log transformed levels in tumor vs. control (p = 0.001).

All these markers except APRIL, IFN-β and TWEAK, were upregulated in tumor samples compared to controls. The log-transformed levels which were normally distributed but exhibited no difference between tumor tissue and control were those of CD30 (significance value p = 0.115).

**Figure 2:**
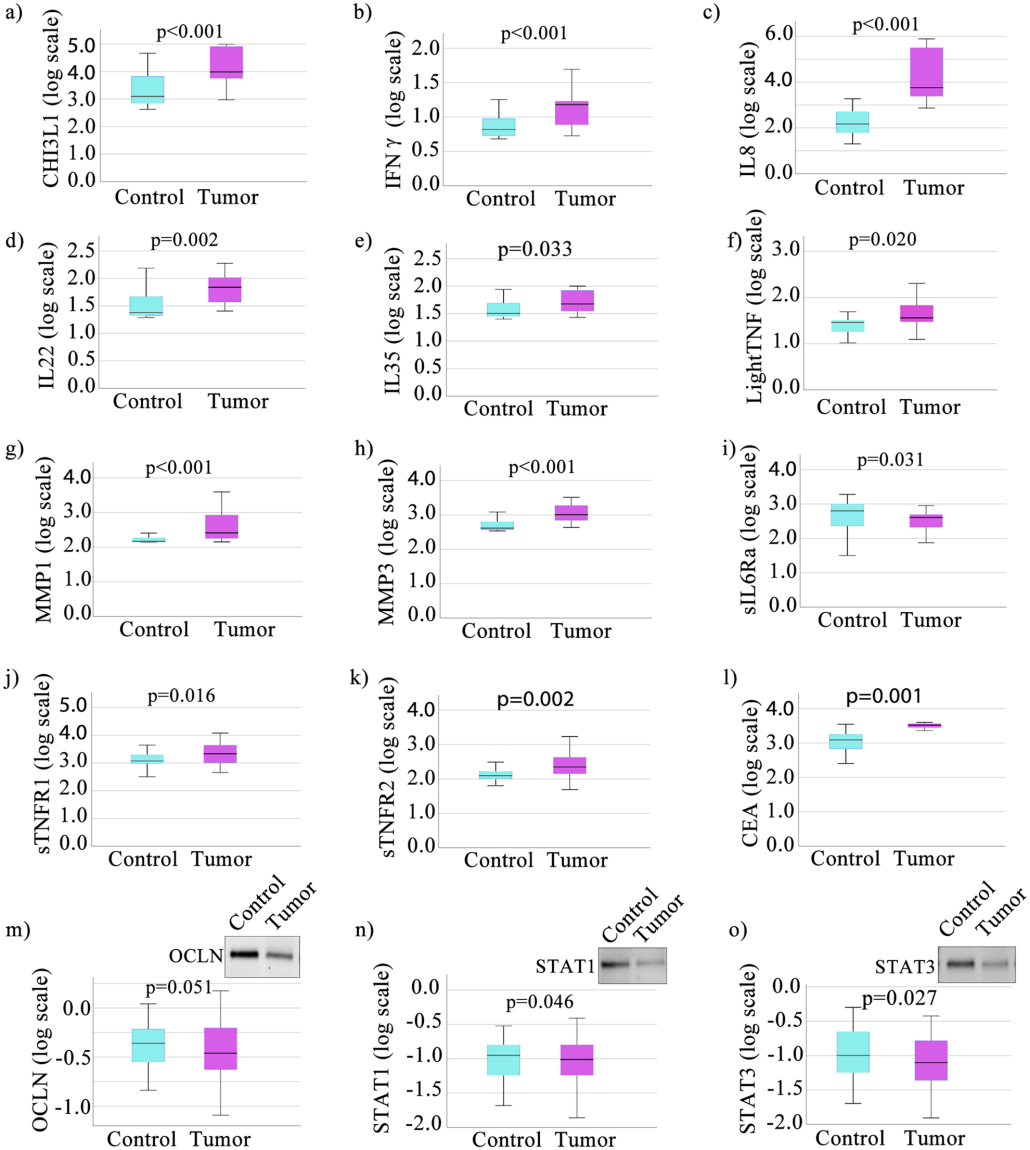
Box-plots of log-transformed biomarker levels in tumor and paired normal tissue samples. Selected log-transformed biomarker values did not exibit normal distribution. (**a**) CHI3L1; (**b**) IFN γ; (**c**) IL8; (**d**) IL22; (**e**) IL35; (**f**) Light TNF; (**g**) MMP1; (**h**) MMP3; (**i**) sIL6Rα; (**j**) sTNFR1; (**k**) sTNFR2; (**l**) CEA; (**m**) OCLN; (**n**) STAT1; (**o**) STAT3; in box-plots (**m**), (**n**), (**o**) the source data was the average relative density of immunoblot bands of all tumor and paired normal tissue samples; the cropped inserts show representative immunoblot examples for OCLN, STAT1, STAT3, The original immunoblots are available in Supplementary Figures S2, S3 and S4; *p *values resulting from comparison of control and tumor means of log-transformed concentration values through the Wilcoxon test are written above each boxplot pair.

The log-transformed levels which do not exhibit a normal distribution but exhibit statistically significant tumor vs. control differences via Wilcoxon tests were (in alphabetical order): CEA, Chitinase 3-like 1 (CHI3L1), IFN-γ, IL-8, IL-22, IL-35, LIGHT/TNFSF14, OCLN, MMP-1, MMP-3, sIL-6Rα, STAT1, STAT3, sTNFR1 and sTNFR2 (Fig. 2). All these markers except sIL-6Rα, OCLN, STAT1 and STAT3, are upregulated in tumor samples compared to controls. Additionally to the biomarkers presented in Fig. 2, we found the downregulation of CP (p = 0.050) and the upregulation of gp130 (p = 0.050), IL-20 (p = 0.003), IL-32 (p = 0.006), IL-34 (p = 0.033) and osteopontin (OPN) (p < 0.001). For protein carbonyl immunoblot examples, see Supplementary Figure S5. The log-transformed values which were not normally distributed and exhibited no differences between tumors and controls were those of Pentatrexi3 (p = 0.390) and that of CD163 (p = 0.123).

### Histopathology and immunohistochemistry

After assessing hematoxylin and eosin slides in order to evaluate the immune infiltrate levels, our samples fell into three qualitative categories: reduced (54.5%), moderate (41%) and large (4.5%) immune infiltrate. Moreover, we have noticed that CD163 protein expression ratios (tumor/control) were sample-wise positively correlated to immune infiltrate level in hematoxilin and eosin (HE) slides i.e. reduced immune infiltrates were correlated with low CD163 ratios (0.0–0.5), moderate immune infiltrate with intermediate CD163 ratios (0.5–4.0) and large immune infiltrate with CD163 ratios (4.0–6.0). We did not have sufficient data to verify if such a correlation can be generalized to other conditions (e.g. other types of solid tumors or other CRC patient cohorts). Samples have been also analyzed by immunohistochemistry and tested for microsatellite instability. All the tumors have been found microsatellite stable.

### Correlation matrix

**Figure 3:**
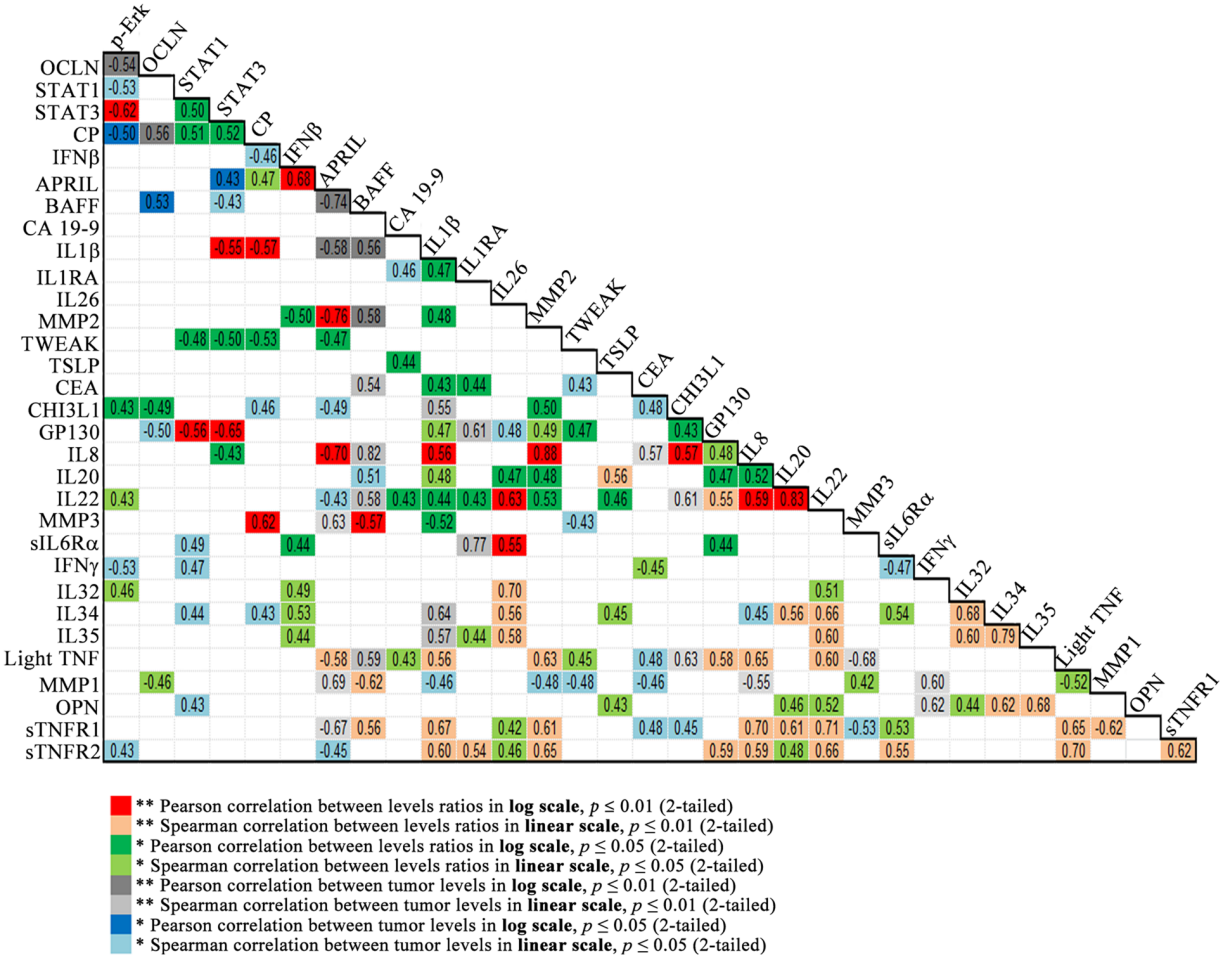
Combined multi-type variables correlation matrix containing the highest values of the correlation coefficients. Combinations of the two levels of significance (0.01 and 0.05) and the type of correlation (Pearson or Spearman) is color-coded, as shown in the legend below the matrix.

A new set of variables has been defined as the ratio between the biomarker levels in tumors and in control samples. Correlations between biomarker levels and ratios were gathered into a combined multi-type variables correlation matrix (see Fig. 3). This matrix has been compiled by reporting the most significant correlation coefficients as follows: log-transformed biomarker levels ratios which were normally distributed are represented by Pearson correlation coefficients, the untransformed (linear scale) biomarker level ratios are represented by Spearman coefficients, the log-transformed biomarker tumor levels which are normally distributed are represented by Pearson coefficients, and untransformed (linear scale) biomarker tumor levels are represented by Spearman coefficients.

We note five positive correlation coefficients greater than 0.751: MMP2-IL8 (concentrations ratios in log scale) = 0.884** (Pearson), BAFF-IL8 (concentrations in tumors) = 0.820** (Spearman), IL20-IL22 (concentrations ratios in log scale) = 0.833** (Pearson), IL34-IL35 (concentration ratios in log scale) = 0.792** (Spearman) and IL6Rα-IL1RA (concentrations in tumors) = 0.772** (Spearman).

Also, we note the strongest five negative correlations (less than − 0.650): APRIL–MMP2 (concentration ratios in log scale) = − 0.761 (Pearson), APRIL-BAFF (concentrations in log scale) = − 0.740** (Pearson), APRIL-IL8 (concentrations ratios in log scale) = − 0.696** (Pearson), MMP3-LIGHT/TNFSF14 (concentrations in tumor) = − 0.684** (Spearman) and APRIL-sTNFR1 (concentrations in tumor) = − 0.667** (Spearman).

**Figure 4:**
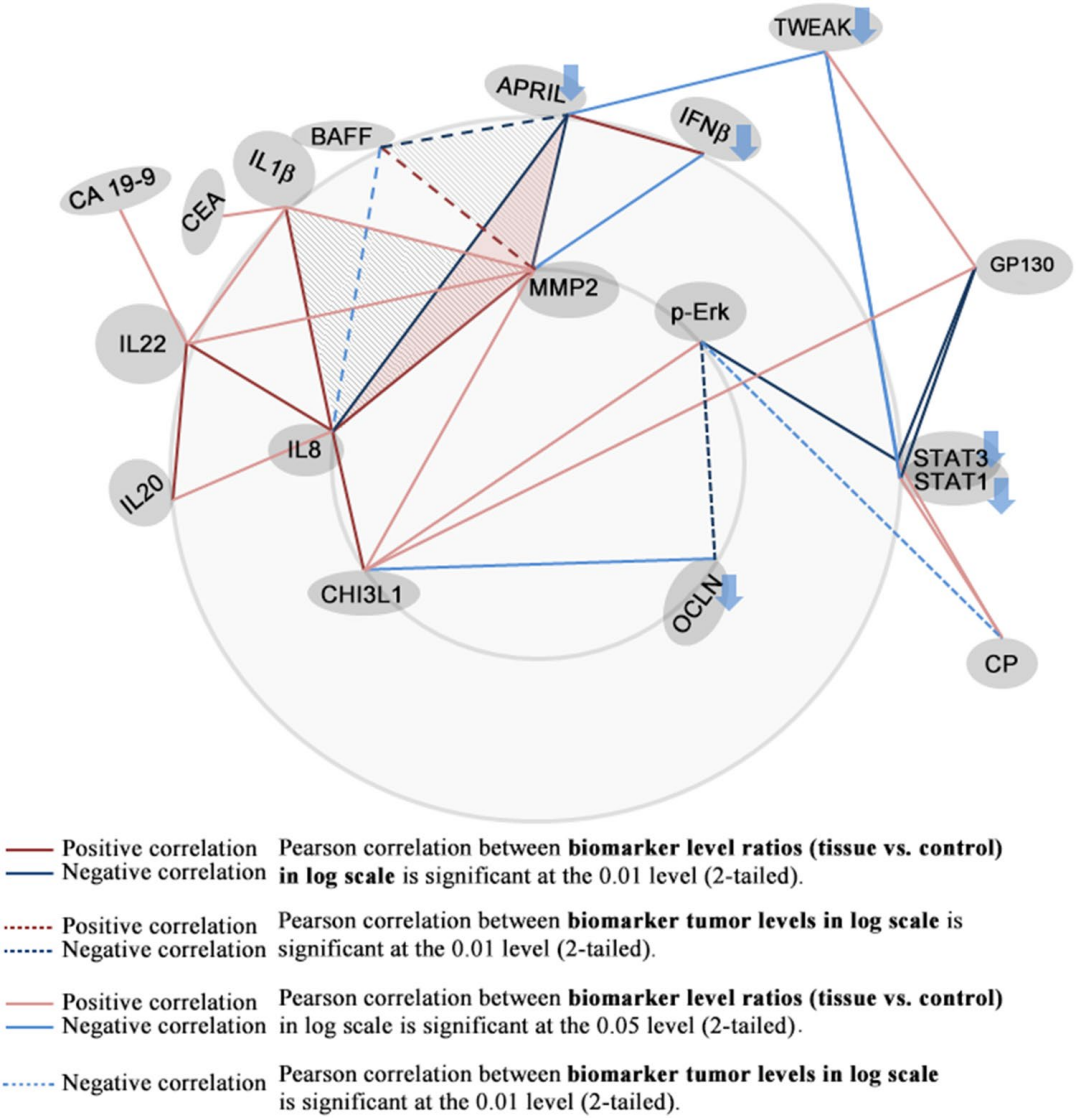
Correlated biomarkers pairs connected according to their two by two positive (in red) or negative (in blue) Pearson correlation significance levels. All Pearson correlation values are shown in the Fig. 3 correlation matrix.

Selected biomarkers pairs with positive Pearson correlation coefficients (ranging between 0.47 and 0.83) and negative Pearson correlation coefficients (ranging from − 0.76 and − 0.48) (as shown in Fig. 3) have been grouped in clusters represented as triangles and quadrangles (Fig. 4) with the sides, representing the actual two-by-two correlations, drawn using different color-coded types of lines corresponding to the type of variables (biomarker levels ratio or biomarker tumor levels, in linear or log scale).

**Figure 5:**
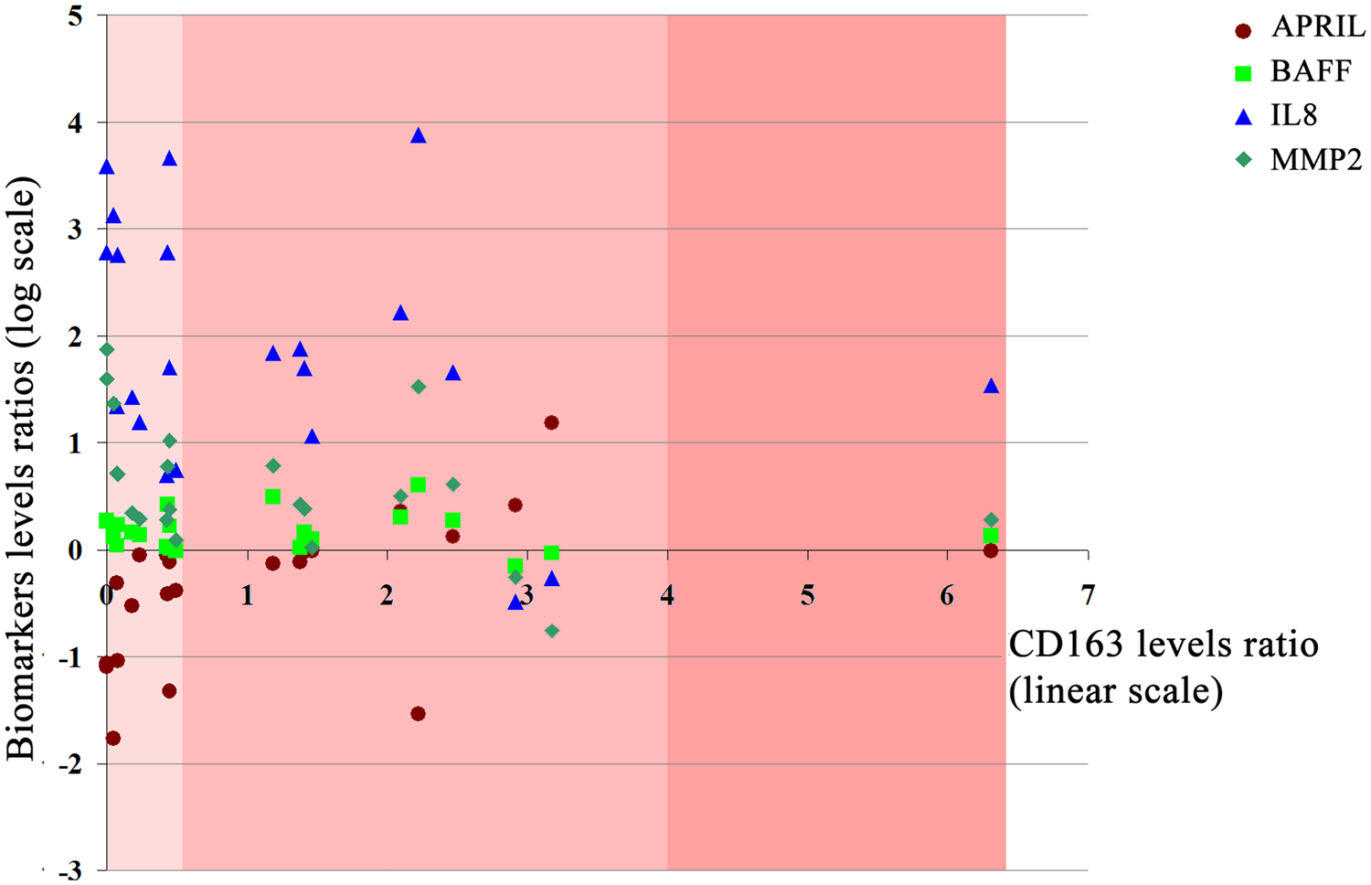
Plot of APRIL, BAFF, IL8 and MMP2 ratios (tumor/normal tissue) in log scale versus the CD163 ratios (tumor/normal tissue). Linear trend lines sharing the same color as the data points have been represented for each data set. The scatter plot chart area has been divided into three regions corresponding to the CD163 ratios 0.001–0.5, 0.5–4.0 and 4.0–6.0, with increasing red color intensity corresponding to the immune infiltrate observed in the histopathology reports: reduced, moderate and large.

The four biomarkers levels ratios (tumor/control) involved in the strongest above-mentioned correlations i.e. APRIL, BAFF, IL8 and MMP2 were patient-wise negatively correlated to the CD163 levels ratios (Fig. 5).

For the normally distributed biomarkers (presented in Fig. 1) demonstrating reliable strong correlations, we have plotted their absolute levels and grouped the samples according to the tumor AJCC stages (I, II, III, IV) with their corresponding paired normal tissue controls (see Supplementary Figure S6). An Independent-Samples Kruskal–Wallis Test has been applied to the biomarker tumor levels in order to find any possible differences across staging groups. The test has been applied to all these biomarkers and also to IL-8 which was involved in several strong correlations highlighted in Fig. 4. With the exception of IL 26 (p = 0.044), there was no statistically significant difference in tumor biomarker expression across the groups containing samples of the same stage (Supplementary Table S2).

## Discussion

### Underexpressed and low level biomarkers

An important number of cytokine levels were below the detection limit of the method (LOD and/or LLOQ) such as IL-2, IL-10, IL-11, IL-12 (p40), IL-12 (p70), IL-19, IL-27 (p28), IL-28A/IFN-λ2, IL-29/IFN-λ1, IFN-α2 and osteocalcin in both tumor and normal tissue control samples. Most of these cytokines were previously reported as participants in the regulation of the anti-tumor immune response. These findings might suggest the existence of a pro-malignant phenotype in these CRC patients. Another potential explanation for such downregulation is that these cytokines are active mainly during very early stages of tumor formation. This group of cytokines deserves more attention in the future because it might reveal potent biomarkers for early stage tumor detection. For example, IL-27, whose concentrations were below our method detection limit, was previously shown to posess a potent antitumor activity, related not only to the induction of tumor-specific Th1 and cytotoxic T lymphocyte responses but also to direct inhibitory effects on tumor cell proliferation, survival, invasiveness, and angiogenic potential^14^. In addition, IL-27 together with IL-12 (another undetectable marker in our samples) mediates the activation of both STAT1 and STAT3 and also it can enhance CD4+T cell proliferation, Th1 cell differentiation, and IFN-γ production^14^. Moreover, IFN-*γ* production was low in both normal and tumor tissue (Supplementary Table S1) and STAT1 and STAT3 were downregulated in tumor versus normal tissue (Fig. 2n,o). Hence, IL-27 and his co-enhancing partner IL-12 may not be active in the stages were neoplastic tissue is well differentiated into a tumor, as it is in the case of our cohort formed by patients with tumors beyond the IIA stage.

Both IFN-α and IFN-β, together with IL-28 have been reported to have potent antitumor activity and are currently used in the clinical treatments of several malignancies^15^. Our results show that IFN-α and IL-28 are poorly expressed and IFN-β is significantly downregulated in tumors compared to controls (Fig. 1c).

Finally, we emphasize the low IL-2 levels in our samples. The antitumor effect of high-dose IL2 therapy was demonstrated more than three decades ago^16^. Now, IL2 is used in cancer immunotherapy for the expansion of immune cells such as NK cells, T-cells, NKT-cells, cytokine-induced killer cells and is also used as an adjuvant in the treatment of patients with melanoma, advanced colorectal cancer or ovarian cancer with autologous dendritic cells stimulated by autologous tumor lysate^6^. The efficacy of these therapies is easy to understand as the suppressed IL-2 levels are a barrier in cellular immune response.

### CHI3L1-related pathways

CHI3L1 has been proposed both as a biomarker and a potential therapeutic target in gastric and colorectal cancer, being overexpressed in serum and tumor tissue^17–20^ being involved in promoting cancer cell proliferation, invasion and metastasis^21^. As shown in Fig. 2a, the protein expression levels in cancer tissues were significantly higher than in adjacent normal tissues of the same patients (p < 0.001). Our results are in accordance with the CHI3L1 plasma levels observed in CCR patients *vs.* control and mRNA expression levels detected by Kawada et al. in colorectal cancer tissue versus normal tissues^17^. In addition, their results revealed that a strong CHI3L1 expression was significantly correlated with T stage, lymphatic invasion, vascular invasion and lymph node metastasis and the in vitro studies on SW480 cells indicated that CHI3L1 overexpression induces IL-8 secretion and up-regulation of Erk and JNK pathway^17^. In our cohort, immunoblot analysis of pErk1/2 (Fig. 1j) revealed that this pathway is active, the pErk1/2 levels were significantly increased in tumor tissue compared to control (p < 0.001). Moreover, the MMP-2 and IL-8 levels (Figs. 1g, 2c) were significantly higher in tumor tissue vs. control (p < 0.001). Our results showed a significant Pearson correlation coefficient between CHI3L1 and pERk (0.43) on the one hand and between CHI3L1 and MMP2 (0.503) on the other hand (Figs. 3, 4). Also, a strong Pearson correlation coefficient (0.59) was found between CHI3L1 and IL8 (Figs. 3, 4). The CHI3L1–Erk correlation is in agreement with the previously proposed mechanisms involved in tumor biology. Thus, CHI3L1 binding to CD44v3 induces the activation of Erk, Akt, and β-catenin signaling pathways enhancing cancer metastasis^22^. In cell lines, CHI3L1 stimulation results in the phosphorylation of Erk1/2^23^ and a recombinant CHI3L1 was reported to significantly enhance the proliferation of SW480 cells, through the activation of MAPK/Erk signaling pathway^24^. Overall, CHI3L1-induced MMP2 overexpression plays a crucial role in ECM regulation promoting cancer cell growth, proliferation, invasion, and metastasis^24^. The CHI3L1, MMP2, and IL8 form a triad (Fig. 4) based on the correlation matrix (Fig. 3), which seems to play a central role in tumor local and distal development.

Another important triad correlation (Fig. 4) revealed the link between the upregulation of pErk (Fig. 1a) and CHI3L1 (Fig. 2a) and the downregulation of OCLN (Fig. 2m). The possible involvement of the Ras-Raf-MEK-ERK signaling module in regulating OCLN expression was previousley examined in Pa-4 cells transiently transfected with either an oncogenic K-RAS, an active mutant of Raf-1, Raf BXB or with constitutively active MEK1, pFC-MEK1. In all these transfected cells, northern blots revealed decreased OCLN mRNA levels. In addition, downregulation of OCLN expression induced by pFC-MEK1 was blocked by PD98059, a selective inhibitor of Erk activation. Furthermore in A549 cell model, with a high Ras-Raf signaling activity due to an oncogenic K-ras mutation, an upregulation of OCLN was demonstrated by transfecting a dominant negative Raf-1 construct or by treating the cells with PD98059^25^. These results are in agreement with our study showing that elevated pErk levels are inversely correlated with OCLN protein expression (Pearson correlation − 0.54; p < 0.01), reinforcing that the decrease in OCLN is due to the activation of the Erk signaling pathway. Moreover, 45% of patients were diagnosed with K-RAS mutation (data not shown). In addition, a Pearson correlation coefficient (− 0.49) was determined between CHI3L1 and OCLN (Fig. 3).

Selected biomarkers pairs with positive Pearson correlation coefficients (ranging between 0.47 and 0.83) and negative Pearson correlation coefficients (ranging from − 0.76 and − 0.48) (as shown in Fig. 3) have been grouped in clusters represented as triangles and quadrangles (Fig. 4) with the sides representing the actual two-by-two correlations. Corresponding to the type of variables (biomarker levels ratio and biomarker tumor levels in linear and log scales) for which the correlation has been calculated, the sides have been drawn using different color-coded types of lines (as explained in the legend).

### IL-8 an important cross-link point in cytokines constellation

We emphasize the fact that in our tumor samples IL-8 cytokine had the highest levels vs. controls (fourfold increase, Fig. 2c, Supplementary Table S1). Our study showed that this highly expressed biomarker is a member of a highlighted correlation triad formed by IL-8, IL-β1 and MMP-2 with Pearson correlation coefficients of 0.56, 0.48 and 0.88, respectively (Figs. 3, 4). In agreement with the IL-8 and MMP2 positive correlation revealed by our data, Pengjun et al. (2013) showed that compared with the healthy controls, the colorectal adenoma patients exhibited a concomitant increase of IL-8 and MMP-2^26^. Consecutively, the importance of MMP-2 was demonstrated by Groblewska et al.^27^ who showed that positive tissue expression of MMP-2 was a significant prognostic factor for CRC patients survival being involved in the invasion and metastasis of CRC.

An important implication in EMT was also revealed by IL-22 in relation with IL-1β^28^, which was shown to be critically involved in CRC cell metastasis^29^. We showed an important correlation between IL-8, IL-1β, MMP-2 and IL-22 (Figs. 3, 4). IL-22 has tumor-promoting properties, enhances tumor-cell proliferation, protects against apoptosis, and mediates the attraction of immunosuppressive immune cells and the release of other pro- and anti-inflammatory cytokines^30^. In addition, cancer cells induce IL-22 production by memory CD4+T cells via IL-1β in order to promote tumor growth^31^.

A study of the immune condition of CRC patients^1^ has suggested a direct relation between IL-8 and CA19-9 on one hand and IL-8 and CEA on the other hand. Our study suggests that such relation might develop through intermediary correlations such as IL8–IL22–CA19-9 and IL8–IL1 β–CEA, respectively (see Fig. 4).

As discussed above, β-catenin pathway activation through IL-8 induces EMT and the increases of MMP-2 expression and activity^32^. We found a downregulation of IFN-β in tumor tissue compared to control (Fig. 1c) and a negative Pearson correlation with MMP-2 (− 0.501). Moreover, in CRC it was demonstrated that β-catenin could inhibit the expression of IFN-β and interferon-stimulated gene 56 (ISG56) by interacting with the central transcription factor, interferon‐regulatory factor 3, blocking its nuclear translocation, responsible for the induction of IFN‐β and hence being essential for the activation of interferon responses^33^. This result might be relevant for immunotherapy development because it has recently been shown that IFN-β can sensitize CRC cells to 5-fluorouracyl treatment with a potent effect on the reduction of tumor mass, suggesting a novel strategy to selectively target CRC^34^.

### APRIL, BAFF, IL-8 and MMP-2 cluster as potential therapeutic targets

Only scarce data exist about APRIL and BAFF roles in CRC tumor biology. The breast cancer is one of the few solid tumors described to display a differential role of BAFF and APRIL, which are produced in important quantities either by stromal cells or infiltrating neutrophils^35^. BAFF is constantly present in both normal and tumor tissue, while APRIL is preferentially expressed by the non-cancerous breast epithelial tissue, while its expression was shown to be decreased in breast tumor cells. In addition, APRIL expression was inversely correlated with the tumor stage of breast cancers^35,36^.

Recently the possible role of APRIL–BAFF and of their receptors in solid tumors has been studied by using Oncomine resources to investigate The Cancer Genome Atlas (TCGA) in order to compare tumor vs. normal mRNA expression in the whole spectrum of the samples collection. All the tumor samples analyzed exhibited APRIL and BAFF expression, together with those of their receptors. APRIL and BAFF were usually downregulated in tumors as compared to normal control. CRC was the only exception: BAFF is upregulated in tumor samples as compared to their non-transformed counterparts^36^. Data from over 881 CRC tissue samples were analyzed and these reports come in support of our results, that show one of the strongest negative Pearson correlation between APRIL and BAFF (− 0.740). Additionally, our results also showed strong correlation between BAFF and IL8 (0.820, Spearman), APRIL and BAFF (− 0.740, Pearson), APRIL and IL8 (− 0.696, Pearson), APRIL and MMP2 (− 0.761, Pearson), MMP2 and IL8 (0.884, Pearson).

The levels ratios (tumor vs. control) of APRIL, BAFF, IL-8 and MMP-2 were patient-wise negatively correlated in log scale with CD163 protein expression ratios (tumor/control) (as shown in Fig. 5). CD163 is a potential inflammation biomarker, which can be found either free (sCD163) or bound to the plasma membrane of macrophage cells^37^, and it is not known to be expressed in the normal colon tissue^38^. The CD163 protein tissue level can thus be a good indicator of the presence of active inflammatory foci. Moreover, CD163 has also been reported to bind TWEAK^37^ that has a downregulated expression in tumor tissues vs. control (Fig. 1). The immune infiltrate levels (reduced, moderate or large) in HE slides relate in a patient-wise manner to the CD163 expression levels ratio: reduced immune infiltrate is correlated with low values of the CD163 ratio while large immune infiltrate is related with high values of the CD163 ratio. This suggests that the four marker cluster might be expressed mostly by the CRC tumor cells and less by the immune infiltrate cells.

### The STAT 1 and STAT3 downregulation plays a role in tumor growth

The STAT1 and STAT3 transcription factors have been identified as major players in tumor genesis and they are being considered promising targets for cancer therapy. They are thought to play opposite roles, namely, STAT1 is involved in activating immunosurveillence and it is considered a tumor suppressor^39^, while STAT 3 is considered an oncogene, and its persistent signaling contributes to stimulate cell proliferation and prevent apoptosis^2^. STAT1 inhibition was reported in diverse tumor types, along with overexpressed STAT3^40^. However, the role of STAT3 in CRC development remains controversial, as there are reports that show elevated STAT3 had tumor promoting activity and also tumor inhibitory effects^41^. Interestingly, more recently, concomitant absence of STAT1 and STAT3 was reported in CRC tumor tissue, which was found to be significantly correlated with shorter overall survival of CRC patients^41^. In vitro experiments on various colon cancer cell lines revealed that STAT3 activity is subjected to particular changes in the inflammatory tumor microenvironment^42^, most notably IL6 high levels stimulate STAT3.

In vitro functional studies revealed that IL6 utilizes the GP130/IL6 hexameric signaling complex, which includes IL6Rα^43^, a receptor chain component which is typically upregulated in cancer cell lines. Here we report, both diminished levels of STAT1 and STAT3 in CRC as compared to non-transformed colon tissue, but also reduced IL6Rα, which may contribute to explain this phenomenon. Interestingly, recent developments of therapeutic approaches are considering the pharmacological blockage of STAT3 signaling^43^, however, given our findings of already reduced STAT1 and 3 in CRC tumors and also the association of low STAT1 concomitant with STAT3 with reduced survival of CRC patients^41^, great caution should be taken when designing such therapies.

As a major final by-product of multiple oxidation pathways that occur in the cell, protein carbonylation is an appropriate marker of oxidative stress. Modification of the carbonylated protein levels is associated with the pathogenesis of several diseases, including colorectal cancer (CRC)^44^. The current understanding researchers in this field share is that activated oncogenes (specifically K-Ras^G12D^, B-Raf^V619E^ and Myc^ERT2^) contribute to enhance the transcription Nrf2, leading to a more intense antioxidant program, that in turn contributes to decrease tumor cell reactive oxygen species (ROS) levels^45^. In support of this idea, our findings indicate that protein carbonylation levels significantly decreased in CRC tumor tissue. This may also contribute to explain why such perturbations of redox homeostasis could favor worse outcomes in CRC. Interestingly, we found positive correlations between CP and both STAT1 and STAT3 (Figs. 3, 4). Although there is tentative data suggesting that in some tumor cells both STAT1 and STAT3 post-transcriptional regulation are stimulated by ROS^46^, and specifically STAT3 phosphorylation can be activated^47^, there is insufficient data regarding colon cancer STAT1/3 ROS regulation, and this may be a good direction for further study as it could contribute to elucidate the role of ROS in CRC, and potentially clarify the controversial role of antioxidants in cancer treatment.

Finally, in the series of down regulated biomarkers related to STAT1 and STAT3, we mention TWEAK. In CRC patients, increased tumor levels of TWEAK were found to be associated with statistically significantly higher overall survival^48^. In vitro invasion assays revealed TWEAK displaying an inverse relation with tumor invasive ability. Our study reveals significantly reduced TWEAK in tumor samples, which is negatively correlated in tumor tissues with MMP1 and MMP3 (Figs. 3, 4).

This study is a systematic investigation of a wide range of inflammatory factors involved in colon cancer related signaling pathways. We report the existence of a panel of underexpressed markers [namely IL-2, IL-10, IL-11, IL-12 (p40), IL-12 (p70), IL-19, IL-27 (p28), IL-28A/IFN-λ2, IL-29/IFN-λ1, IFN-α2] in CRC patients, which should be further studied to reveal their role in establishing a pro-tumoral inflammatory conditions. Our results bring into focus an overlooked association of markers which might be specific to CRC, namely diminished APRIL levels and high BAFF, as opposed to other tumor types which display both APRIL and BAFF downregulated levels. Together with IL-8 and MMP2, these two markers show strong correlation. Our results show that, APRIL, BAFF, IL8 and MMP2 expression levels are patient-wise inversely related to the immune infiltrate level. Moreover, in the case of these four biomarkers expressions in tumor, no statistically significant differences have been found across the staging groups. New treatments could be developed based on these four biomarkers related biosignature expression pattern.

## Methods

### Study design

This study was performed in accordance with the Declaration of Helsinki 1975, amended in 2013. All protocols and methods carried out were reviewed and approved by the Medical Ethics Committee of Elias University Emergency Hospital in Bucharest (no: 5939/2019). Prior to being included in the study, written informed consents have been signed by all participant patients.

Our patient cohort included 28 patients who underwent surgery in order to remove colorectal tumors at Elias University Emergency Hospital between January 2019 and May 2020. However, 6 of these patients have tested positive for SARS-CoV2, the day after surgery. Since at biological level, the inflammatory profile of these patients might have been influenced by this viral infection known to trigger the so-called “cytokine storm”^49^ these 6 patients have been excluded from the study. The remaining *n* = *22* patients have been included in the study and submitted to the study protocol described below.

In Table 1, we present the demographic and clinical data of our study cohort. The cohort is formed 54.5% (12) male and 45.5 (10) female subjects. Body Mass Index (BMI) of the whole cohort has been categorized according to the World Health Organization classification of obesity^50^. Although both obesity and CRC incidence rates are increasing, it remains unclear what relationship, if any, exists between BMI, cancer recurrence and patient survival^51^. We have defined the following categories of tumor localization according to the colorectal anatomic segments: proximal region and distal region.

**Table 1:** Clinical, demographic and staging data of the study cohort.

Variable	Mean	Median	SD	Min	Max
**Age (years)**	66.14	64.50	9.285	50	81
Female	64.90	64.00	8.412	50	81
Male	67.17	66.50	10.205	52	81
**BMI (kg/m**^**2**^)	27.19	26.57	4.426	20	41
Female	28.36	27.08	5.644	20	41
Male	26.22	26.40	3.011	22	32

Variable	Category	Value	%		
Gender	Female	10	45.5		
	Male	12	54.5		
BMI (kg/m^2^)	Underweight (< 18.5)	0	0		
	Normal range (18.5–24.9)	8	36.4		
	Overweight (≥ 25)	14	73.6		
	Pre-obese (25.0–29.9)	10	45.4		
	Obese class 1 (30.0–34.9)	3	13.7		
	Obese class 2 (30.0–34.9)	0	0		
	Obese class 3 (≥ 40)	1	4.5		
Smoking habits	Smokers	5	22.7		
	Female	1	4.5		
	Male	4	18.2		
	Non-smokers	17	77.3		
	Female	11	50.0		
	Male	6	27.3		
Arterial hypertension	Optimal (TAs < 120 and TAd < 80)	2	9.2		
	Normal (TAs = 120–129 and/or TAd = 80–84)	14	63.6		
	High normal (TAs = 130–139 and/or TAd = 85–89)	5	22.7		
	Grade 1 (TAs = 140–149 and/or TAd = 90–99)	1	4.5		
Staging	I	2	9.1		
	IIA	6	27.3		
	IIB	2	9.1		
	IIIB	3	13.6		
	IIIC	3	13.6		
	IVA	6	27.3		

Within this definition 40.9% of patients (9) were subjected to surgery, concerning tumors localized in the distal region and 59.1% of patients (13) were subjected to surgery concerning tumors situated in the proximal region.

Prior to the surgery the patients have been submitted to standard arterial tension measurements. The mean value allowed the classification of these patients according to the European Society of Cardiology (ESC) and the European Society for Hypertension (ESH) criteria^52^ in optimal, normal, high normal blood pressure (BP) and grade 1–3 hypertension. Table 1 shows that most of the patients 72.7% (16) had optimal or normal BP while 27.2% (6) of them were in the early stages of arterial hypertension (high normal BP and grade 1 stages).

All samples have been assessed by HE staining, in order to establish the staging and to evaluate the immune infiltrate. The cancer staging in Table 1 has been established using the ypTNM classification of tumors from histopathological samples by AJCC classification criteria grid^53^. We have classified our samples into three relatively broad qualitative categories: reduced, moderate and large immune infiltrate. For the purpose of treatment initiation, all patients have been submitted to MSI analysis through immunohistochemistry in private specialized laboratories outside of Elias University Emergency Hospital.

### Tissue samples and lysates preparation

This study analyzed tumor and paired normal tissue samples, available from 22 patients who underwent surgery in order to remove colorectal tumors. Tumor tissue and adjacent normal mucosa from each patient were excised and immediately frozen at − 80 °C until analysis. Frozen tumor and paired normal tissue (100 mg) were thawed and homogenized in ice cold cell lysis buffer (provided by Bio-Plex Pro cell signaling kit, Bio-Rad Laboratories, Hercules, CA, USA) containing a protease inhibitor cocktail using ceramic beads and TeSeE PRECESS 24 Homogenizer (Bio-Rad Laboratories, Hercules, CA, USA) according to the manufacturer's instructions. The lysate was placed on ice for 10 min and then centrifuged at 14,000×*g* for 10 min. The protein content of the supernatant was determined for each sample using the Pierce Rapid Gold BCA Protein Assay Kit (Thermo Fisher Scientific, MA, USA). Finally, the supernatant was aliquoted and frozen at − 80 °C, for subsequent biochemical and immunochemical analyses.

### Biomarkers quantization

#### Inflammatory cytokines and MMPs

Supernatant samples with a total protein concentration of 900 μg/mL were used for the detection of APRIL/TNFSF13, BAFF/TNFSF13B, sCD30/TNFRSF8, sCD163, CHI3L1, gp130/sIL-6Rβ, IFN-α2, IFN-β, IFN-γ, IL-2, sIL-6Rα, IL-8, IL-10, IL-11, IL-12 (p40), IL-12 (p70), IL-19, IL-20, IL-22, IL-26, IL-27 (p28), IL-28A/IFN-λ2, IL-29/IFN-λ1, IL-32, IL-34, IL-35, LIGHT/TNFSF14, MMP-1, MMP-2, MMP-3, Osteocalcin, Osteopontin, Pentraxin-3, sTNF-R1, sTNF-R2, TSLP and TWEAK/TNFSF12 biomarkers using the Bio-Plex Pro Human Inflammation 37-plex Panel1 (Bio-Rad Laboratories, Hercules, CA, USA). The IL-1β and IL-1Ra was quantified separately using the Bio-Plex Pro Human Cytokine Standard 27-plex, Group I, magnetic beads and detection antibodies for human IL-1β and IL-1RA (Bio-Rad Laboratories, Hercules, CA, USA). The biomarker levels were analyzed according to manufacturer’s instructions, using the Bio-Plex MAGPIX System and Bio-Plex Manager software version 6.0 (Bio-Rad Laboratories, Hercules, CA, USA) as previously described^54,55^.

### ELISA assay for CEA and CA19-9

CEA and CA19-9 levels from tissue lysates were quantified by using CanAg CEA EIA kit (Fujirebio Diagnostics AB, Sweden) and CanAg CA19-9 EIA 120–10 (Fujirebio Diagnostics AB, Sweden), according to the manufacturer’s protocol. The protein concentration of the tissue lysate for CEA assay was 1.5 mg/mL and for CA19-9 assay was 75 μg/mL.

### Western blot assays

For target protein expression evaluation, protein tissue lysates were resolved on Protean TGX Stain Free 4%–20% precast gels (Bio-Rad Laboratories, Hercules, CA, USA), transferred onto 2 μm nitrocellulose membrane (V3 Western Workflow, Bio-Rad Laboratories, Hercules, CA, USA) and total protein transferred signal was detected and quantified using the ChemiDoc MP System and Image Lab software (version 5.2.1, Bio-Rad Laboratories, Hercules, CA, USA). The membranes were blocked using EveryBlot Blocking Buffer for 5 min at room temperature. Mouse anti-human ERK/MAPK (pThr202/pTyr204) monoclonal antibody (MCA5990GA, 1:1000 dilution factor), mouse anti-human OCLN monoclonal antibody (MCA3308Z, 1:250 dilution factor), mouse anti-human STAT1 monoclonal antibody (MCA3469Z, 1:350 dilution factor) and goat anti-human STAT3 polyclonal antibody (AHP1076, 1:500 dilution factor), were used. HRP conjugated secondary antibodies (STAR 120 and STAR 122 at 1:500 and 1:15.000 dilution factor respectively, Bio-Rad antibodies, Hercules, CA, USA) were used. For immunostaining of the membranes, they were incubated with the primary antibodies for 2 h and with the secondary antibodies for 1 h at room temperature, under constant homogenization. Blots were revealed using the Clarity Western ECL Substrate (Bio-Rad Laboratories, Hercules, CA, USA) and the chemiluminescence signal was detected using the ChemiDoc MP System. The target proteins expression was quantified using the Image Lab software version 5.2.1 (Bio-Rad Laboratories, Hercules, CA, USA), and normalized to the total proteins transferred onto the membrane (each protein band was normalized against the total proteins transferred in the corresponding lane)^56^.

Cellular carbonylated protein detection was performed using the OxiSelect Protein Carbonyl Immunoblot Kit (Cell Biolabs, San Diego, CA, USA) with a post-transfer derivatization step of protein-bound carbonyl groups by treatment with a solution of dinitrophenylhydrazine (DNPH). The formed adducts were recognized by a primary rabbit anti-DNPH (diluted 1:1000), and HRP conjugated secondary antibody anti-rabbit IgG (diluted 1:1000) as previously described^54^.

### Statistical analysis

#### Preliminary data validation

The biomarkers levels were analyzed with Bio-Plex Manager software version 6.0 (Bio-Rad Laboratories, Hercules, CA, USA). The coefficient of variation (COV), percentage recovery and normality distribution of data was checked before proceeding to the statistical analysis. Data distribution for sample replicates has been assessed using COV with15% intra-assay precision and 20% inter-assay precision.

To ensure the data reliability quality controls have been run in parallel to the Bio-Plex sample assay. These assay controls are in fact samples containing analytes in known concentration provided by the assay manufacturer. Percentage recovery of 80–120% is considered acceptable to support accurate sample interpretation.

#### Statistical analysis steps

After the validation of COV and percentage step, statistical analysis was performed as follows: normality checks, log transformations for data normalization, comparisons control vs. tumor samples and correlations. Data analysis has been performed using the IBM SPSS Statistics 26 statistical analysis package.

Our first objective is to assess the statistically significant differences for all markers in control vs. tumor samples by applying the corresponding tests. The first step in choosing the comparison test is to check the normality distribution of the raw concentration and densitometry data.

The Shapiro–Wilks test (p > 0.05) and visual inspection of their histograms, normal Q–Q plots and box plots have been used at this stage to check the normality distribution for each biomarker concentrations data for both control and tumor sample. In the Shapiro–Wilks test the null hypothesis is the normal distribution of data. This hypothesis is rejected for p < 0.05 and it is accepted for p > 0.05.

Data sets which failed the initial normality test i.e. for which *p* < 0.05, have been normalized through log transformation i.e. *y* → log (*y*). The newly obtained data sets have been again submitted to the Shapiro–Wilks normality test.

Our samples were paired-related samples being collected from the same patients according to the above described protocol. The normally distributed data sets have been submitted to parametric means comparison testing (t Student tests, see Fig. 1) and the data sets which were not normalized post log transformation have been submitted to non-parametric comparison testing (Wilcoxon, see Fig. 2). The biomarkers concentrations were considered statistically significant distinct if *p* < 0.05. The biomarkers which failed the comparison tests have been excluded.

For the purpose of correlation, a new set of variables have been defined as the ratio between the biomarker level in tumor and that in control. The levels ratios normal distribution has been checked by Shapiro–Wilks testing. The ratios failing the first stage normality testing have been log-transformed and the Shapiro–Wilks test has been applied again.

Log-transformed variables and ratios that are normally distributed have been included in Pearson correlation matrices, while the remaining variables and the ratios that were not normally distributed have been included in Spearman correlation matrices. Since all the untransformed variables and ratios were not normally distributed, the Spearman correlation coefficients were also calculated for both tumor related data and ratios.

The correlation coefficients represent a measure of a monotonic relation between the paired data. The nearer this coefficient is to ± 1 the stronger the monotonic relationship is. If the correlation is significant at a 0.01 level (2-tailed) the coefficient value is marked by two stars (**) and if this correlation is significant at a 0.05 level the coefficient value is marked by a star (*).

All the correlation matrices between untransformed tumor related data, log-transformed normally distributed variables, log transformed normally distributed ratios and the untransformed ratios have been associated to a combined multi-type variables correlation matrix (see Fig. 3). The correlation coefficients have been colored specifically to identify their origin and a hierarchy has been established according to the following criteria: (1) Pearson correlations were considered stronger than Spearman correlations, (2) 0.01 (2 tailed) coefficients are stronger than the 0.05 (2 tailed) coefficients and (3) ratios related correlations were considered stronger than untransformed simple variable correlations.

All normally distributed biomarkers and IL-8 which was not normally distributed but exhibited high upregulation in tumor with respect to control have been submitted to an Independent-Samples Kruskal–Wallis Test to check across the staging groups for any statistically significant differences in the biomarker expression in tumor tissue.

## Supplementary Information

Supplementary Information

## References

[1] Komura T (2018). Immune condition of colorectal cancer patients featured by serum chemokines and gene expressions of CD4+ cells in blood. Can. J. Gastroenterol. Hepatol..

[2] Corvinus FM (2005). Persistent STAT3 activation in colon cancer is associated with enhanced cell proliferation and tumor growth. Neoplasia.

[3] Klampfer L (2011). Cytokines, inflammation and colon cancer. Curr. Cancer Drug Targets.

[4] Coussens LM, Werb Z (2002). Inflammation and cancer. Nature.

[5] Nogueira-Costa G (2020). Prognostic utility of neutrophil-to-lymphocyte ratio in patients with metastatic colorectal cancer treated using different modalities. Curr. Oncol..

[6] Chulpanova DS, Kitaeva KV, Green AR, Rizvanov AA, Solovyeva VV (2020). Molecular aspects and future perspectives of cytokine-based anti-cancer immunotherapy. Front. Cell Dev. Biol..

[7] West NR, McCuaig S, Franchini F, Powrie F (2015). Emerging cytokine networks in colorectal cancer. Nat. Rev. Immunol..

[8] Vainer N, Dehlendorff C, Johansen JS (2018). Systematic literature review of IL-6 as a biomarker or treatment target in patients with gastric, bile duct, pancreatic and colorectal cancer. Oncotarget.

[9] Xu J (2016). Diagnostic and prognostic value of serum interleukin-6 in colorectal cancer. Medicine (Baltimore).

[10] Akhmaltdinova L (2020). Inflammatory serum biomarkers in colorectal cancer in Kazakhstan population. Int. J. Inflamm..

[11] Bhardwaj M (2020). Multiplex screening of 275 plasma protein biomarkers to identify a signature for early detection of colorectal cancer. Mol. Oncol..

[12] Nikolaou S (2018). Systematic review of blood diagnostic markers in colorectal cancer. Tech. Coloproctol..

[13] Annaházi A (2016). A pilot study on faecal MMP-9: A new noninvasive diagnostic marker of colorectal cancer. Br. J. Cancer.

[14] Fabbi M, Carbotti G, Ferrini S (2017). Dual roles of IL-27 in cancer biology and immunotherapy. Mediators Inflamm..

[15] Numasaki M (2007). IL-28 elicits antitumor responses against murine fibrosarcoma. J. Immunol..

[16] West WH (1989). Continuous infusion recombinant interleukin-2 (rIL-2) in adoptive cellular therapy of renal carcinoma and other malignancies. Cancer Treat Rev..

[17] Kawada M (2012). Chitinase 3-like 1 promotes macrophage recruitment and angiogenesis in colorectal cancer. Oncogene.

[18] Faibish M, Francescone R, Bentley B, Yan W, Shao R (2011). A YKL-40-neutralizing antibody blocks tumor angiogenesis and progression: A potential therapeutic agent in cancers. Mol. Cancer Ther..

[19] Johansen JS (2008). Elevated plasma YKL-40 predicts increased risk of gastrointestinal cancer and decreased survival after any cancer diagnosis in the general population. J. Clin. Oncol..

[20] Johansen JS (2015). Serum YKL-40 in risk assessment for colorectal cancer: A prospective study of 4,496 subjects at risk of colorectal cancer. Cancer Epidemiol. Biomark. Prev..

[21] Qiu Q-C (2018). CHI3L1 promotes tumor progression by activating TGF-β signaling pathway in hepatocellular carcinoma. Sci. Rep..

[22] Geng B (2018). Chitinase 3-like 1-CD44 interaction promotes metastasis and epithelial-to-mesenchymal transition through β-catenin/Erk/Akt signaling in gastric cancer. J. Exp. Clin. Cancer Res..

[23] Areshkov PO, Avdieiev SS, Balynska OV, Leroith D, Kavsan VM (2012). Two closely related human members of chitinase-like family, CHI3L1 and CHI3L2, activate ERK1/2 in 293 and U373 cells but have the different influence on cell proliferation. Int. J. Biol. Sci..

[24] Zhao T, Su Z, Li Y, Zhang X, You Q (2020). Chitinase-3 like-protein-1 function and its role in diseases. Signal Transduct. Target Ther..

[25] Li D, Mrsny RJ (2000). Oncogenic Raf-1 disrupts epithelial tight junctions via downregulation of occludin. J. Cell Biol..

[26] Pengjun Z (2013). Multiplexed cytokine profiling of serum for detection of colorectal cancer. Future Oncol..

[27] Groblewska M (2014). Serum levels and tissue expression of matrix metalloproteinase 2 (MMP-2) and tissue inhibitor of metalloproteinases 2 (TIMP-2) in colorectal cancer patients. Tumour Biol..

[28] Baker KJ, Houston A, Brint E (2019). IL-1 family members in cancer; two sides to every story. Front. Immunol..

[29] Ieda T (2019). Visualization of epithelial-mesenchymal transition in an inflammatory microenvironment-colorectal cancer network. Sci. Rep..

[30] Huber S (2012). IL-22BP is regulated by the inflammasome and modulates tumorigenesis in the intestine. Nature.

[31] Voigt C (2017). Cancer cells induce interleukin-22 production from memory CD4+ T cells via interleukin-1 to promote tumor growth. Proc. Natl. Acad. Sci..

[32] Wen J (2020). IL-8 promotes cell migration through regulating EMT by activating the Wnt/β-catenin pathway in ovarian cancer. J. Cell Mol. Med..

[33] Ding C (2018). β-catenin regulates IRF3-mediated innate immune signalling in colorectal cancer. Cell Prolif..

[34] Di Franco S, Turdo A, Todaro M, Stassi G (2017). Role of Type I and II interferons in colorectal cancer and melanoma. Front. Immunol..

[35] Pelekanou V (2008). Expression of TNF-superfamily members BAFF and APRIL in breast cancer: Immunohistochemical study in 52 invasive ductal breast carcinomas. BMC Cancer.

[36] Kampa M, Notas G, Stathopoulos EN, Tsapis A, Castanas E (2020). The TNFSF members APRIL and BAFF and their receptors TACI, BCMA, and BAFFR in oncology, with a special focus in breast cancer. Front. Oncol..

[37] Etzerodt A, Moestrup SK (2013). CD163 and inflammation: Biological, diagnostic, and therapeutic aspects. Antioxid. Redox Signal.

[38] Krijgsman D (2020). CD163 as a biomarker in colorectal cancer: The expression on circulating monocytes and tumor-associated macrophages, and the soluble form in the blood. Int. J. Mol. Sci..

[39] Avalle L, Pensa S, Regis G, Novelli F, Poli V (2012). STAT1 and STAT3 in tumorigenesis: A matter of balance. JAKSTAT.

[40] Buettner R, Mora LB, Jove R (2002). Activated STAT signaling in human tumors provides novel molecular targets for therapeutic intervention. Clin. Cancer Res..

[41] Nivarthi H (2018). The ratio of STAT1 to STAT3 expression is a determinant of colorectal cancer growth. Oncotarget.

[42] Quante M, Varga J, Wang TC, Greten FR (2013). The gastrointestinal tumor microenvironment. Gastroenterology.

[43] Thilakasiri P (2019). Repurposing the selective estrogen receptor modulator bazedoxifene to suppress gastrointestinal cancer growth. EMBO Mol. Med..

[44] Hawk MA, McCallister C, Schafer ZT (2016). Antioxidant activity during tumor progression: A necessity for the survival of cancer cells?. Cancers (Basel).

[45] DeNicola GM (2011). Oncogene-induced Nrf2 transcription promotes ROS detoxification and tumorigenesis. Nature.

[46] Butturini E, CarcereridePrati A, Mariotto S (2020). Redox regulation of STAT1 and STAT3 signaling. Int. J. Mol. Sci..

[47] Qu Y (2013). Generation of prostate tumor-initiating cells is associated with elevation of reactive oxygen species and IL-6/STAT3 signaling. Can. Res..

[48] Lin B-R (2012). Prognostic significance of TWEAK expression in colorectal cancer and effect of its inhibition on invasion. Ann. Surg. Oncol..

[49] Ragab D, Salah Eldin H, Taeimah M, Khattab R, Salem R (2020). The COVID-19 cytokine storm; what we know so far. Front. Immunol..

[50] Aronne LJ (2002). Classification of obesity and assessment of obesity-related health risks. Obes. Res..

[51] Lee D-W, Cho S, Shin A, Han S-W, Kim T-Y (2020). Body mass index and body weight change during adjuvant chemotherapy in colon cancer patients: Results from the AVANT trial. Sci. Rep..

[52] Williams B (2018). 2018 ESC/ESH Guidelines for the management of arterial hypertension: The Task Force for the management of arterial hypertension of the European Society of Cardiology (ESC) and the European Society of Hypertension (ESH). Eur. Heart J..

[53] *AJCC Cancer Staging Manual*, (Springer International Publishing, 2017).

[54] Geicu OI (2020). Dietary AGEs involvement in colonic inflammation and cancer: Insights from an in vitro enterocyte model. Sci. Rep..

[55] Serban AI, Stanca L, Geicu OI, Dinischiotu A (2015). AGEs-induced IL-6 synthesis precedes RAGE Up-regulation in HEK 293 cells: An alternative inflammatory mechanism?. Int. J. Mol. Sci..

[56] Geicu OI, Stanca L, Dinischiotu A, Serban AI (2018). Proteomic and immunochemical approaches to understanding the glycation behaviour of the casein and β-lactoglobulin fractions of flavoured drinks under UHT processing conditions. Sci. Rep..

