# Supplementary material for: Key biomarkers within the colorectal cancer related inflammatory microenvironment
Source: Sci Rep. 2021 Apr 12;11:7940. doi: 10.1038/s41598-021-86941-5 (PMC8041790; doi:10.1038/s41598-021-86941-5)
Supplement: Supplementary file 1 — Supplementary Information [file 41598_2021_86941_MOESM1_ESM.docx]

**Key biomarkers within the colorectal cancer related inflammatory microenvironment**

**Valentin Calu^1,2^, Adriana Ionescu^3^, Loredana Stanca^4^, Ovidiu Ionut Geicu^4,5^, Florin Iordache^4^, Aurelia Magdalena Pisoschi^4^, Andreea Iren Serban^3,4^*, Liviu Bilteanu^4,6^**

^1^Department of General Surgery, University of Medicine and Pharmacy “Carol Davila” Bucharest, 8 Blvd., Eroii Sanitari, 050474, Bucharest, Romania.

^2^Department of Surgery "Elias" Emergency University Hospital, 17 Marasti Blvd., 01146, Bucharest, Romania.

^3^Department of Biochemistry and Molecular Biology, Faculty of Biology, University of Bucharest, 91-95 Blvd. Splaiul Independentei, 050095, Bucharest, Romania.

^4^Department of Preclinic Sciences, Faculty of Veterinary Medicine, University of Agronomic Sciences and Veterinary Medicine of Bucharest, 105 Blvd. Splaiul Independentei, 050097, Bucharest, Romania.

^5^ Taxon Solutions SRL, No.7 Semilunei, 020797, Bucharest, Romania.

^6^National Institute for Research and Development in Microtechnologies, No. 126A Erou Iancu Nicolae, 077190, Bucharest, Romania

*Correspondence should be addressed to A.I.S. (email: irensro@yahoo.com).

**Supplementary Table 1**. Some descriptive statistics values of marker concentrations higher than LOD and exhibiting statistically significant differences in tumor and control samples.

| **Biomarker** | **LOD**  **(pg/mL)** | **Control** | | | | | | **Tumor** | | | | | |
| --- | --- | --- | --- | --- | --- | --- | --- | --- | --- | --- | --- | --- | --- |
|  |  | **Minimum** | **25% Percentile** | **Median** | **Mean** | **75% Percentile** | **Maximum** | **Minimum** | **25% Percentile** | **Median** | **Mean** | **75% Percentile** | **Maximum** |
| **APRIL** | 190.0 | 968.2 | 9660.8 | 27962.1 | 41778.6 | 60010.7 | 187097.2 | 949.5 | 2196.8 | 9096.5 | 23674.5 | 38773.9 | 94616.5 |
| **BAFF** | 34.7 | 1071.7 | 1838.7 | 2782.6 | 3373.8 | 4577.5 | 7343.9 | 2250.9 | 3033.4 | 4789.0 | 4709.3 | 6099.6 | 8188.3 |
| **CHI3L1*** | 10.3 | 422.9 | 665.6 | 1250.0 | 7252.8 | 6799.9 | 46247.8 | 937.4 | 5563.6 | 9723.7 | 35301.5 | 83633.2 | 98800.0 |
| **GP130** | 16.9 | 61.2 | 235.4 | 3531.1 | 3795.1 | 4640.5 | 25643.6 | 64.5 | 491.2 | 3502.7 | 4674.8 | 6832.0 | 22635.9 |
| **IL1b** | 0.3 | 0.6 | 3.8 | 9.0 | 27.3 | 15.1 | 320.2 | 3.0 | 12.9 | 36.1 | 592.7 | 191.7 | 9875.3 |
| **IL1RA** | 2.2 | 127.3 | 326.1 | 720.9 | 933.2 | 1634.3 | 2610.1 | 120.5 | 461.2 | 1039.7 | 1561.9 | 1893.6 | 7127.7 |
| **IFN-β** | 2.0 | 2.6 | 5.1 | 6.6 | 7.7 | 7.8 | 19.1 | 2.6 | 3.7 | 5.5 | 5.5 | 6.8 | 11.4 |
| **IFN-γ** | 0.05 | 4.9 | 5.3 | 6.6 | 8.2 | 9.6 | 17.9 | 5.3 | 7.7 | 15.2 | 14.4 | 16.9 | 49.3 |
| **IL20** | 3.6 | 10.7 | 10.7 | 10.9 | 12.2 | 11.7 | 21.4 | 10.8 | 11.1 | 12.3 | 15.1 | 18.6 | 27.7 |
| **IL22** | 1.1 | 19.6 | 20.8 | 23.8 | 44.4 | 48.0 | 179.9 | 25.5 | 35.6 | 69.2 | 81.1 | 105.1 | 188.0 |
| **IL26** | 1.2 | 57.7 | 195.4 | 255.0 | 324.0 | 542.5 | 673.5 | 157.8 | 232.6 | 406.7 | 423.0 | 559.5 | 772.6 |
| **IL32** | 12.3 | 21.7 | 53.7 | 64.8 | 66.0 | 74.3 | 154.3 | 50.8 | 64.0 | 71.1 | 83.9 | 80.7 | 191.6 |
| **IL34** | 51.9 | 67.9 | 70.5 | 76.0 | 94.0 | 92.3 | 272.7 | 68.3 | 78.0 | 83.5 | 107.1 | 110.7 | 273.4 |
| **IL35** | 3.7 | 25.2 | 28.0 | 31.9 | 47.8 | 55.1 | 138.3 | 27.0 | 34.7 | 47.5 | 57.7 | 85.8 | 100.4 |
| **IL8 *** | 2.7 | 20.2 | 60.8 | 147.9 | 47468.2 | 523.8 | 586200.0 | 739.9 | 2370.0 | 5741.3 | 197597.6 | 369301.5 | 764592.3 |
| **Light/TNF** | 3.1 | 5.8 | 17.2 | 28.9 | 44.4 | 32.5 | 371.0 | 12.4 | 29.6 | 36.3 | 79.7 | 76.9 | 517.9 |
| **MMP1** | 33.7 | 137.7 | 140.4 | 147.4 | 178.9 | 191.1 | 517.9 | 142.3 | 176.8 | 259.3 | 679.3 | 860.2 | 3932.0 |
| **MMP2*** | 39.7 | 1958.4 | 2818.9 | 8597.3 | 16030.7 | 17980.5 | 82302.9 | 4838.4 | 14045.2 | 32317.9 | 71908.0 | 91605.1 | 321819.5 |
| **MMP3** | 28.5 | 343.4 | 370.0 | 414.8 | 598.0 | 709.4 | 1629.6 | 432.8 | 677.9 | 1013.4 | 1354.8 | 1937.0 | 3246.8 |
| **OPN** | 91.3 | 148.3 | 360.9 | 431.1 | 410.8 | 486.6 | 581.6 | 383.1 | 446.4 | 479.5 | 484.2 | 512.6 | 613.7 |
| **sIL6Rα** | 1.5 | 31.6 | 225.3 | 630.5 | 665.9 | 1006.5 | 1903.0 | 33.2 | 200.7 | 405.0 | 387.2 | 500.8 | 901.0 |
| **sTNFR1** | 0.2 | 319.5 | 946.4 | 1197.9 | 3249.4 | 2072.3 | 38591.8 | 452.6 | 1002.8 | 2148.4 | 7119.5 | 4605.8 | 54059.7 |
| **sTNFR2** | 3.2 | 63.4 | 100.3 | 124.5 | 216.5 | 180.8 | 1294.0 | 49.2 | 141.2 | 227.6 | 405.2 | 466.3 | 1722.1 |
| **TSLP** | 0.8 | 2.5 | 7.6 | 9.9 | 9.1 | 11.1 | 13.8 | 4.4 | 7.2 | 8.2 | 8.3 | 9.4 | 11.7 |
| **TWEAK** | 0.5 | 104.7 | 146.6 | 339.2 | 424.7 | 582.1 | 1213.1 | 28.0 | 104.5 | 142.4 | 332.0 | 411.9 | 1689.4 |
| **CA19-9**** | 1.0 | 3.9 | 16.7 | 58.1 | 131.5 | 205.9 | 547.2 | 5.1 | 64.0 | 353.5 | 589.1 | 503.0 | 3027.4 |
| CEA*** | 0.3 | 253.9 | 665.5 | 1230.5 | 1330.9 | 1819.8 | 3529.2 | 613.4 | 2735.1 | 3309.7 | 3052.4 | 3620.0 | 4003.1 |

*The samples that exceeded the ULOQ (upper limit of quantitation) were diluted accordingly.

**CA19-9 units are U/mL.

***CEA units are µg/L

**Supplementary figures**


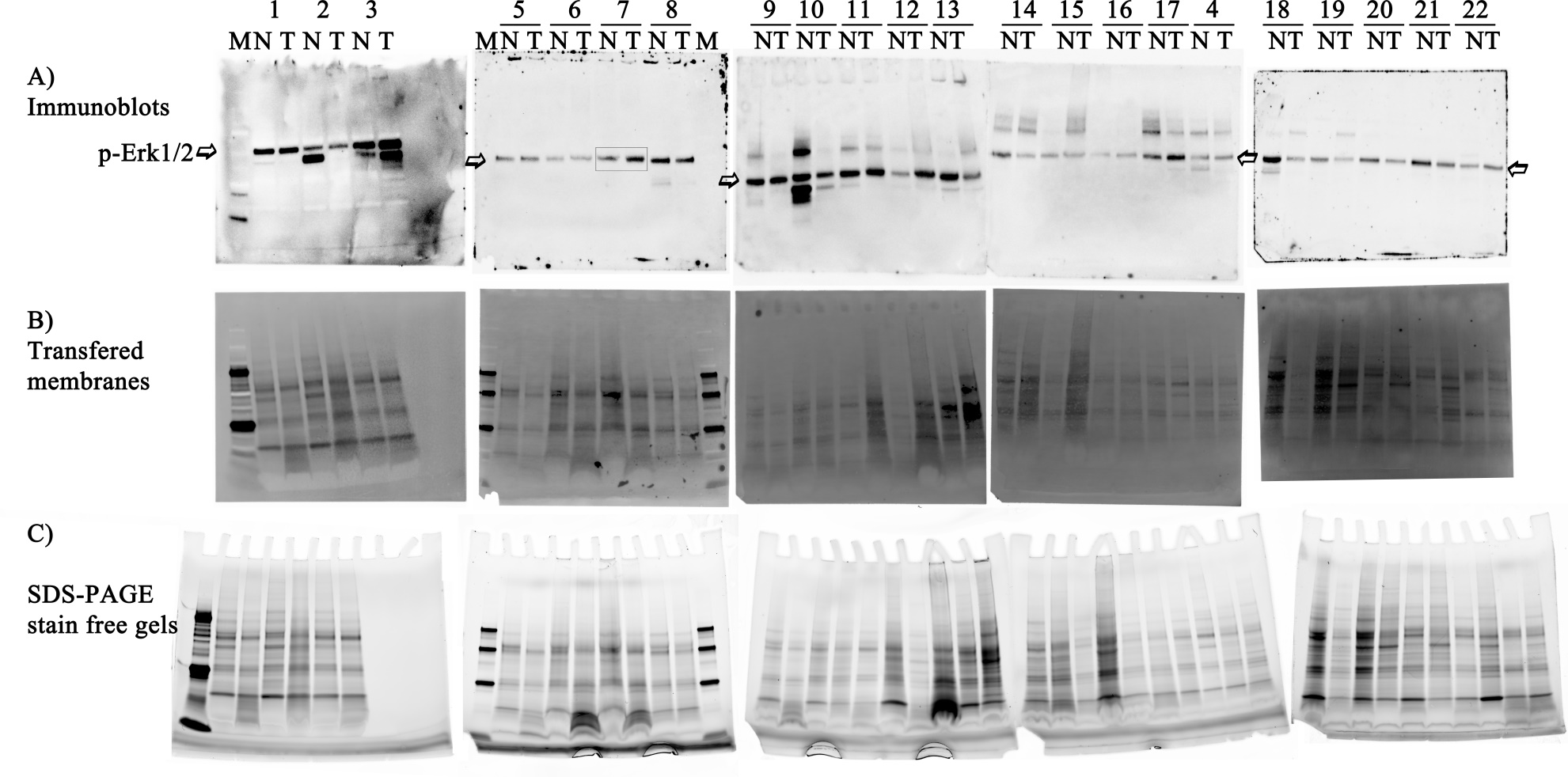


**Supplementary Figure S1. Original whole p-Erk1/2 immunoblot examples** with target protein bands indicated by arrows (A); In-study assigned patient codes are shown at the top of the image with arabic numbers from 1 to 22; from each patient nonstransformed tissue samples (N) and CRC tumoral tissue samples (T) were analyzed, as indicated above each line. A black edged rectangle around the immunoblot bands corresponding to patient 7 is indicating the source of the cropped insert in Fig. 1j. The average normalized densitometry data of three replicate experiments were used for subsequent statistical analysis. M represents the molecular weight marker Precision Plus WesternC (BioRad). The corresponding transfered nitrocellulose membranes are shown below each immunoblot (B); these were used for protein loading normalization of immunoblots. The SDS-PAGE 4-20% stain-free gels corresponding to the transfered membranes are shown in (C).


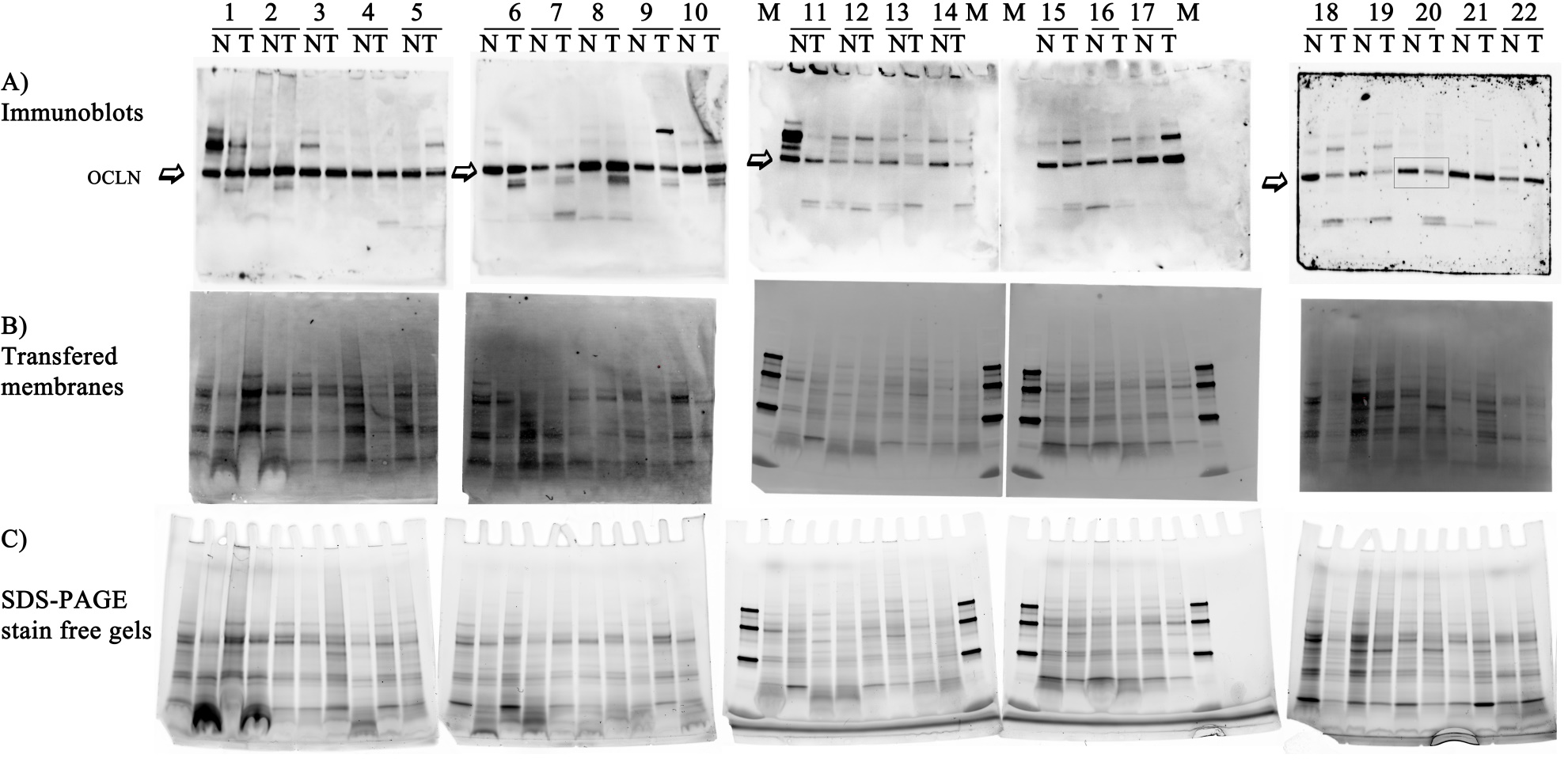


**Supplementary Figure S2. Original whole OCLN immunoblot examples** with occludin bands indicated by arrows (A); In-study assigned patient codes are shown at the top of the image from 1 to 22, and from each patient nonstransformed tissue samples (N) and CRC tumoral tissue samples (T) were analyzed, as indicated above each line. A black edged rectangle around the immunoblot bands corresponding to patient 20 is indicating the source of the cropped insert in Fig. 2m. The average normalized densitometry data of three replicate experiments were used for subsequent statistical analysis. M represents the molecular weight marker Precision Plus WesternC (BioRad). The transfered nitrocellulose membranes are shown in (B), below their corresponding immunoblots. These were used for protein loading normalization of immunoblots. The original, whole SDS-PAGE 4-20% stain-free gels corresponding to the transfered membranes are shown in (C).


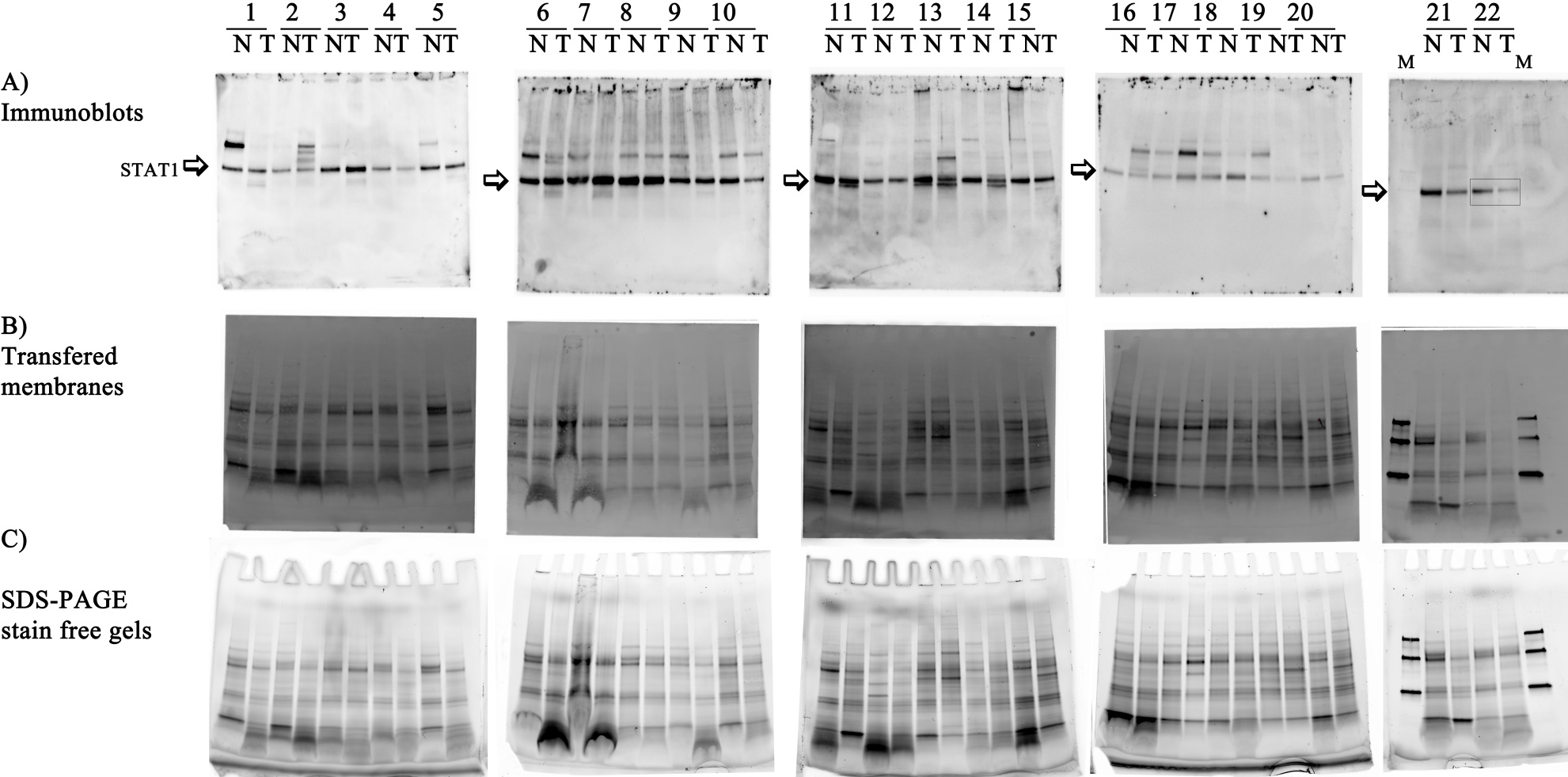


**Supplementary Figure S3**. **Original, whole STAT1 immunoblot examples** with the target protein bands indicated by arrows (A); In-study assigned patient codes are shown at the top of the image, from 1 to 22, and from each patient nonstransformed tissue samples (N) and CRC tumoral tissue samples (T) were analyzed, as indicated above each line. A black edged rectangle around the immunoblot bands corresponding to patient 22 is indicating the source of the cropped insert in Fig. 2n. The average normalized densitometry data of three replicate experiments were used for subsequent statistical analysis. M represents the molecular weight marker Precision Plus WesternC (BioRad). The transfered nitrocellulose membranes corresponding to the immunoblots are shown in (B); These were used for protein loading normalization of immunoblots. The original, whole SDS-PAGE 4-20% stain-free gels corresponding to the transfered membranes are shown in (C).


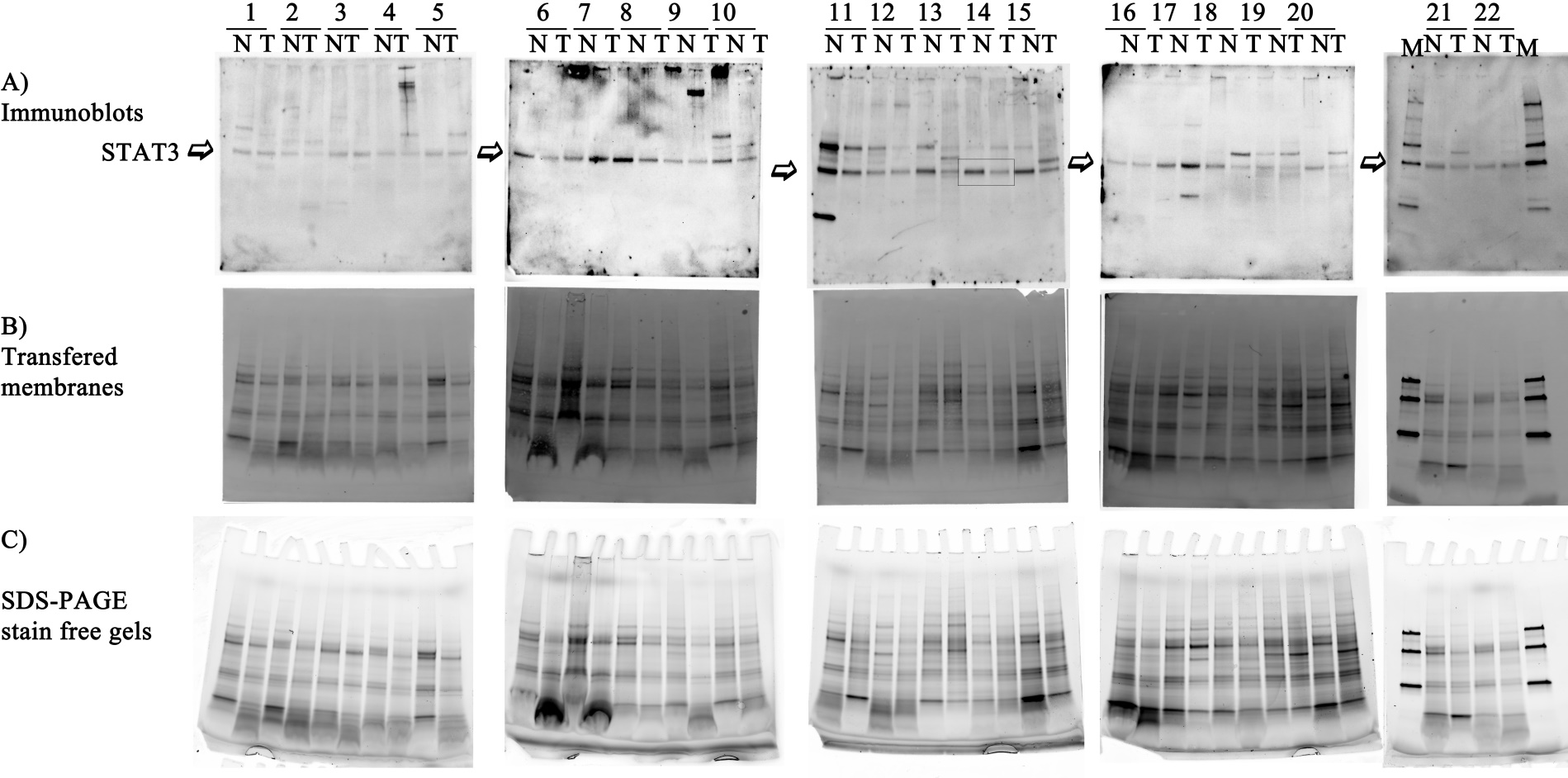


**Supplementary Figure S4. Original, whole STAT3 immunoblot examples** with the target protein bands indicated by arrows (A). In-study assigned patient codes are shown at the top of the image from 1 to 22, and from each patient nonstransformed tissue samples (N) and CRC tumoral tissue samples (T) were analyzed, as indicated above each line. In A) a black edged rectangle around STAT3 protein bands corresponding to patient 14 is indicating the source of the cropped insert in Fig. 2o. The average normalized densitometry data of three replicate experiments were used for subsequent statistical analysis. M represents the molecular weight marker Precision Plus WesternC (BioRad). The transfered nitrocellulose membranes (B) shown below their corresponding immunoblot; these were used for protein loading normalization of immunoblots. The original whole SDS-PAGE 4-20% stain-free gels are shown in (C).


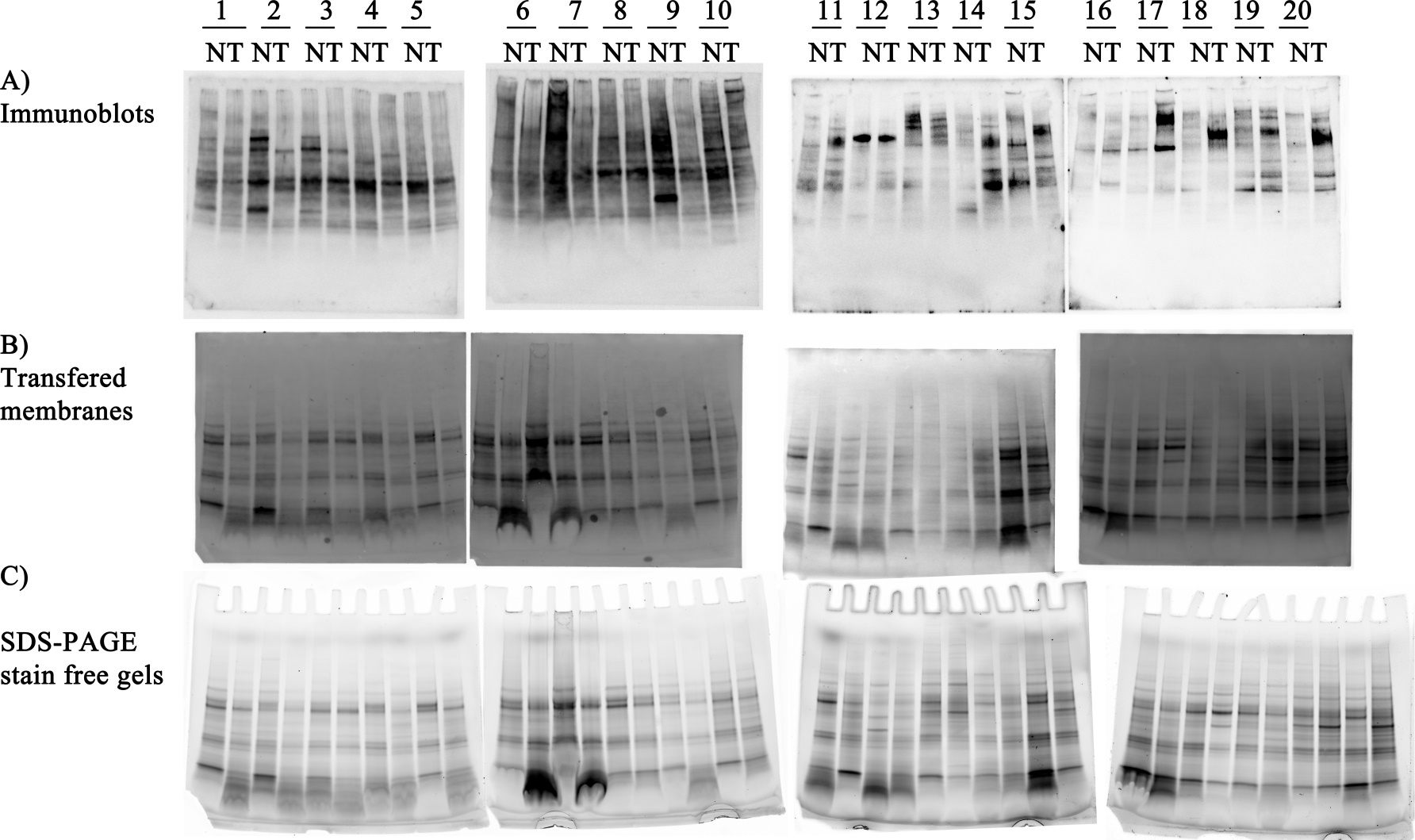


**Supplementary Figure S5. Carbonylated protein immunoblot examples** (A). In-study assigned patient codes are shown at the top of the image from 1 to 20, and from each patient nonstransformed tissue samples (N) and CRC tumoral tissue samples (T) were analyzed, as indicated above each line. M represents the molecular weight marker Precision Plus WesternC (BioRad). Below the immunoblots, their corresponding transfered nitrocellulose membranes are shown in (B). These were used for protein loading normalization of immunoblots. The average normalized densitometry data of three replicate experiments were used for protein carbonyls (PC) subsequent statistical analysis, shown in figure 3. The SDS-PAGE 4-20% stain free gels corresponding to the transfered membranes are shown in (C).


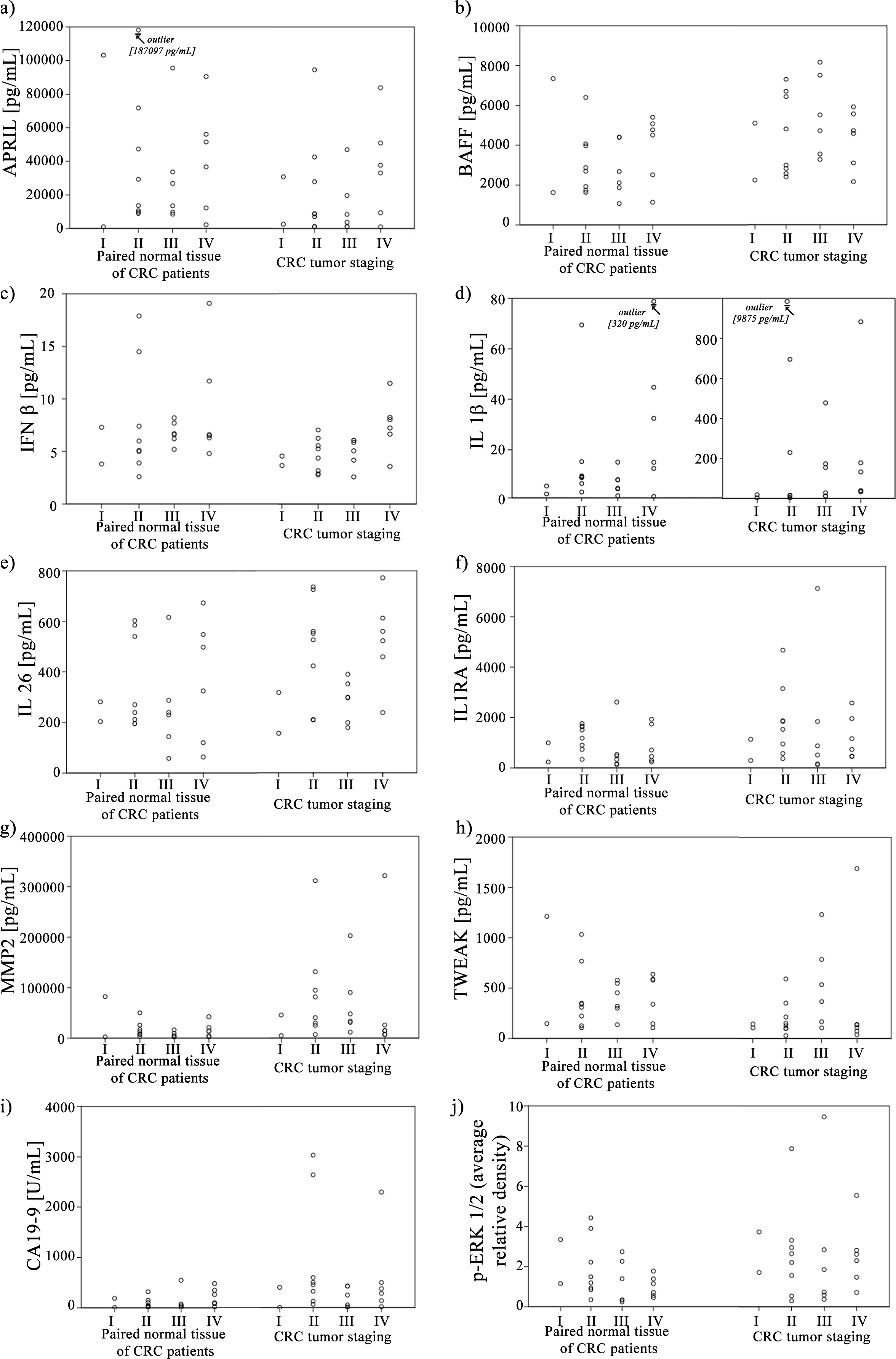


**Supplementary Figure 6.** **Scatterplots of the actual levels in the biomarkers presented in Figure 1.** The data have been grouped by staging (I, II, III, IV). An Independent-Samples Kruskal-Wallis Test has been performed with SPSS Statistics 26 in order to find any possible differences in biomarker levels in tumors across the stages. With the exception of IL 26 (*p=*0.044), there is no statistically significant difference across the groups containing samples of same stage (see Supplementary Table 2).

**Supplementary Table 2**. Independent-Samples Kruskal-Wallis Test Summary. Asymptotic significances are displayed. The significance level is 0.050.

|  | Null Hypothesis | Test | *p* value | Decision |
| --- | --- | --- | --- | --- |
| 1 | The distribution of **APRIL** (tumor) [pg/mL] is the same across categories of Staging. | Independent-Samples Kruskal-Wallis Test | 0.549 | Retain the null hypothesis. |
| 2 | The distribution of **BAFF** (tumor) [pg/mL] is the same across categories of Staging. | Independent-Samples Kruskal-Wallis Test | 0.591 | Retain the null hypothesis. |
| 3 | The distribution of **IL 8** (tumor) is the same across categories of Staging. | Independent-Samples Kruskal-Wallis Test | 0.772 | Retain the null hypothesis. |
| 4 | The distribution of **MMP 2** (tumor) [pg/mL] is the same across categories of Staging. | Independent-Samples Kruskal-Wallis Test | 0.331 | Retain the null hypothesis. |
| 5 | The distribution of IFN β (tumor) [pg/mL] is the same across categories of Staging. | Independent-Samples Kruskal-Wallis Test | 0.076 | Retain the null hypothesis. |
| 6 | The distribution of IL 1β (tumor) [pg/mL] is the same across categories of Staging. | Independent-Samples Kruskal-Wallis Test | 0.335 | Retain the null hypothesis. |
| 7 | The distribution of IL 26 (tumor) [pg/mL] is the same across categories of Staging. | Independent-Samples Kruskal-Wallis Test | 0.044 | Reject the null hypothesis. |
| 8 | The distribution of IL 1RA (tumor) [pg/mL] is the same across categories of Staging. | Independent-Samples Kruskal-Wallis Test | 0.493 | Retain the null hypothesis. |
| 9 | The distribution of TWEAK (tumor) [pg/mL] is the same across categories of Staging. | Independent-Samples Kruskal-Wallis Test | 0.225 | Retain the null hypothesis. |
| 10 | The distribution of CA19-9 (tumor) [U/mL] is the same across categories of Staging. | Independent-Samples Kruskal-Wallis Test | 0.169 | Retain the null hypothesis. |
| 11 | The distribution of p-Erk 1/2 (tumor) [aver. rel. density] is the same across categories of Staging. | Independent-Samples Kruskal-Wallis Test | 0.884 | Retain the null hypothesis. |
